# Respiratory Training and Plasticity After Cervical Spinal Cord Injury

**DOI:** 10.3389/fncel.2021.700821

**Published:** 2021-09-21

**Authors:** Margo Randelman, Lyandysha V. Zholudeva, Stéphane Vinit, Michael A. Lane

**Affiliations:** ^1^Department of Neurobiology and Anatomy, Drexel University College of Medicine, Philadelphia, PA, United States; ^2^Marion Murray Spinal Cord Research Center, Drexel University College of Medicine, Philadelphia, PA, United States; ^3^Gladstone Institutes, San Francisco, CA, United States; ^4^INSERM, END-ICAP, Université Paris-Saclay, UVSQ, Versailles, France

**Keywords:** rehabilitation, spinal cord injury, neuroplasticity, respiration, diaphragm, phrenic

## Abstract

While spinal cord injuries (SCIs) result in a vast array of functional deficits, many of which are life threatening, the majority of SCIs are anatomically incomplete. Spared neural pathways contribute to functional and anatomical neuroplasticity that can occur spontaneously, or can be harnessed using rehabilitative, electrophysiological, or pharmacological strategies. With a focus on respiratory networks that are affected by cervical level SCI, the present review summarizes how non-invasive respiratory treatments can be used to harness this neuroplastic potential and enhance long-term recovery. Specific attention is given to “respiratory training” strategies currently used clinically (e.g., strength training) and those being developed through pre-clinical and early clinical testing [e.g., intermittent chemical stimulation via altering inhaled oxygen (hypoxia) or carbon dioxide stimulation]. Consideration is also given to the effect of training on non-respiratory (e.g., locomotor) networks. This review highlights advances in this area of pre-clinical and translational research, with insight into future directions for enhancing plasticity and improving functional outcomes after SCI.

## Introduction

Respiratory dysfunction is one of the leading causes of morbidity and mortality for individuals with spinal cord injury (SCI) (DeVivo et al., 1993; Winslow and Rozovsky, 2003; Garshick et al., 2005; Hoh et al., 2013). Damage to the neural networks controlling respiration frequently occurs following mid- or high-cervical injuries, which disrupt the phrenic motor circuit. The phrenic network is responsible for diaphragm innervation, which is often considered the primary muscle of respiration (Feldman, 1986; Lane, 2011; Hoh et al., 2013). Therefore, damage to this circuit results in diaphragm paresis or paralysis leading to respiratory deficits (Jackson and Groomes, 1994; Linn et al., 2000). In addition, injuries at this level will at least partially denervate intercostal and abdominal motor pools that are innervated by spinal motor neurons in the thoracic and lumbar spinal cord. The intercostal and abdominal respiratory circuits are also primary respiratory networks that are important for regular inspiratory and expiratory behaviors. Impaired respiratory muscle function can lead to decreased inspiration and vital capacity, potentially complete apnea, ventilator assistance (Jackson and Groomes, 1994; Linn et al., 2000; DiMarco, 2005; Onders et al., 2007), and secondary respiratory complications such as pneumonia (Dalal and DiMarco, 2014). While some spontaneous recovery – or functional plasticity – can occur after injury, it is limited (Vinit et al., 2006; Fuller et al., 2008; Lane et al., 2009), and significant deficits in breathing persist for months post-injury (Fuller et al., 2008; Vinit et al., 2008). There are many methods to assess the extent of these respiratory deficits. These include measures of ventilation, or “breathing behavior” (tidal volume, minute ventilation) and respiratory nerve or muscle activity (diaphragm EMG or phrenic nerve recording) (Lane et al., 2008a).

For the purpose of this review neuroplasticity is defined as the ability of the nervous system to change either anatomically and/or functionally, resulting in persistent alterations in sensorimotor function. These changes can be classified as either beneficial (adaptive plasticity) or detrimental (maladaptive plasticity). While plasticity has been extensively studied during development, learning, and memory, there is a rapidly growing interest in the neuroplastic potential of the injured or degenerating nervous system and how it can be therapeutically harnessed. One prominent example of neuroplasticity after spinal cord injury (SCI) has been documented in the respiratory system with spontaneous functional improvement. Here we summarize experimental as well as clinical evidence for spontaneous respiratory neuroplasticity, discuss methods used to harness this via intentional stimulation of respiratory circuits, and provide a summary of studies that propose mechanisms implicating neurotrophic factors as key players.

## Respiration After Spinal Cord Injury

**FIGURE 1:**
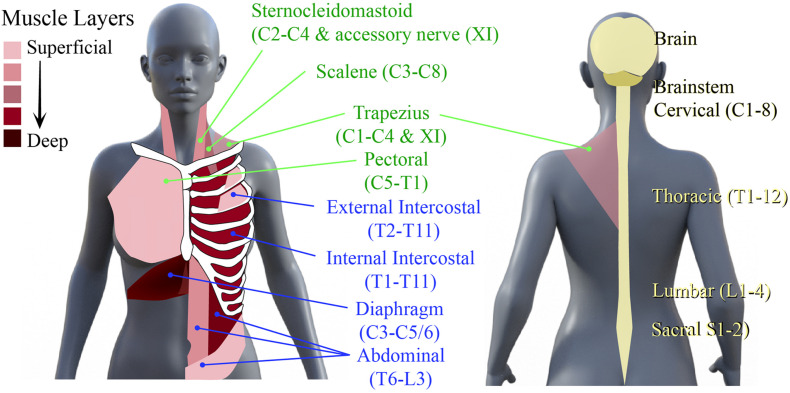
Primary muscles of respiration and their neural circuitry. Schematic diagram of human torso showing the location of the primary muscles of inspiration and expiration and the spinal levels that innervate them.

The neural networks mediating respiratory muscle function, comprising spinal interneurons and lower motoneurons, are distributed throughout the rostro-caudal neural axis. Motoneurons that innervate inspiratory, expiratory, and accessory respiratory muscles can be found throughout the cervical, thoracic, and lumbar spinal cord (Lane, 2011). The primary inspiratory muscles include the diaphragm, external intercostal and scalene muscles, while the primary muscles of expiration are the internal intercostals, rectus abdominals and obliques (Van Houtte et al., 2006; Terson de Paleville et al., 2011). The accessory respiratory muscles, which include the sternocleidomastoid, scalenes, and upper trapezius, are recruited when ventilatory demands are higher than normal (Terson de Paleville et al., 2011; Figure 1). Given the rostro-caudal distribution of these motor networks, injury at any level of the spinal cord can compromise respiratory function. For example, a high cervical SCI usually results in denervation and loss of coordination of all respiratory muscles, leading to quadriplegia and respiratory deficits. This leads to paradoxical movement of the chest walls (De Troyer et al., 1986; De Troyer and Estenne, 1990), decreased pulmonary volumes (Anke et al., 1993; Hopman et al., 1997; Tow et al., 2001) and ineffective cough (Brown et al., 2006; Terson de Paleville et al., 2011). Impaired clearance increases risk of secondary complications such as pneumonia (Brown et al., 2006). Even among those people living with SCI that recover voluntary control of breathing, underlying respiratory deficits persist that can manifest in less overt ways, such as sleep-disordered breathing and episodes of hypoxia.

To treat these deficits respiratory training has been used to stimulate plasticity in networks spared post-SCI. Respiratory training encompasses rehabilitative, resistive, and activity-based training methods to improve and strengthen the neural respiratory circuitry and their corresponding muscles. Early use of respiratory training aimed to strengthen respiratory muscles, using techniques to target inspiratory and expiratory muscles (Figure 1).

## Respiratory Neuroplasticity After Spinal Cord Injury

Spontaneous respiratory neuroplasticity has been reported in both clinical (Hoh et al., 2013) and experimental studies (Mitchell and Johnson, 2003; Goshgarian, 2009; Lane et al., 2009; Nicaise et al., 2013; Warren and Alilain, 2014), serving as an excellent example of how the nervous system adapts to injury in order to maintain a vital, physiological function. While this spontaneous plasticity indicates the neuroplastic potential of the respiratory system, the amount of recovery attributable to this plasticity is limited, and long-term deficits in diaphragm activity persist. While neural plasticity can be used to describe changes in both neuronal and non-neuronal components, neuroplasticity (frequently referred to in this text) usually refers selectively to changes in the neuronal networks (see Box 1 for definitions).

**Defining terminology. This box highlights definitions of terms used throughout this review.**

**Plasticity:** Lasting anatomical and/or functional changes within neural networks or the behaviors they contribute to. These changes usually arise in response to some form of perturbation (e.g., traumatic injury or degenerative disease). Plasticity can also be stimulated or enhanced by increasing activity within these same neural networks (e.g., locomotor training, respiratory training).

**Neural plasticity:** Plasticity within central and peripheral neural networks. This has also been used to encompass the muscles they innervate (neuromuscular plasticity). While neuroplasticity has been used interchangeably with neural plasticity, it can perhaps be more appropriately used to selectively describe changes in the neuronal networks rather than changes in both neuronal and non-neuronal components (neural). Importantly, while plasticity is often thought of as being something that is beneficial, there is a growing appreciation for the fact that plasticity can be adaptive (resulting in beneficial consequences) or maladaptive (resulting in detrimental consequences). An example of the latter would be axonal sprouting and increased connectivity within networks that lead to increased pain or spasticity. For the most part, the plasticity discussed in the present review refers to beneficial types.

**Anatomical neuroplasticity:** Plasticity that typically refers to changes within neuronal connections which can arise via change in synaptic inputs in existing neuronal networks, increased dendritic growth to receive additional inputs, or axonal growth facilitating new neuronal connections. Notably, this neuroplastic axon growth typically arises from collateral sprouts in axonal pathways that were completely spared by injury, from collateral sprouts within injured pathways but proximal to the site of injury, and/or collateral sprouts from injured or non-injured primary afferents. Modest changes can arise spontaneously after injury or be enhanced by treatment.

**Molecular neuroplasticity:** Plasticity that encompasses an altered synthesis of cytokines, such as trophic factors, that can create a plasticity-promoting environment, attracting axons to the appropriate targets (or inappropriate targets in the case of maladaptive plasticity).

**Functional neural plasticity:** The restoration of activity in damaged pathways or increased activity in spared pathways to compensate for damage, which can occur after mild, moderate, and severe SCI (restorative vs. compensatory plasticity).

**Restorative neural plasticity:** Restoration of function in respiratory circuits (and muscles they control) that have been directly compromised/paralyzed by injury.

**Compensatory neural plasticity:** Altered activity within respiratory circuits (and the muscles they control) that are not directly compromised by injury.

**Restorative behavioral neural plasticity:** Restoration of the ability to perform ventilation in exactly the same manner as it was performed prior to injury.

**Compensatory behavioral plasticity:** Effective ventilation, but performed in a manner different from how it was performed prior to injury (e.g., rapid, shallow breathing).

**Maladaptive neural plasticity:** The amplitude or pattern of neural output may become dysfunctional (e.g., weakened or arhythmic), limiting recovery or contributing to deficit.

**Maladaptive behavioral plasticity: Onset of inappropriate patterns of ventilation.**

**Activity based therapy (ABT):** Non-invasive means of increasing motor activity with simultaneous sensory stimulation. In very simple terms this can be thought of as exercise or rehabilitation. Therapeutically, ABTs have been used in both a task specific basis (e.g., training for function within a specific network) or non-task specific basis (e.g., use of respiratory training to improve functional in non-respiratory networks).

**Task-specific training:** Increasing activity or exercise within specific networks to perform a specific task. For instance, training locomotor networks for rhythmic, patterned locomotion versus stance. Similarly, within the respiratory networks, training for breathing under certain conditions may train for activity within networks primarily known to be involved with that activity (e.g., hypoxia vs. hypercapnia). Data from task-specific training, however, needs to be very carefully interpreted as most forms of ABTs can still have off-target effects (e.g., effects on tasks not trained for).

### Anatomical Neuroplasticity

Anatomical respiratory plasticity typically refers to changes within respiratory circuitry, especially neuronal connectivity, that can arise spontaneously after injury or be driven by treatment. Within the spinal cord, there is evidence of spontaneous plasticity involving axonal sprouting, rerouting (Vinit et al., 2005; Vinit and Kastner, 2009; Darlot et al., 2012), and the formation of new polysynaptic connections with phrenic motoneurons via cervical spinal interneurons (Lane et al., 2008b, 2009; Sandhu et al., 2009; Darlot et al., 2012).

One of the most commonly described models of pre-clinical SCI used to study respiratory plasticity has been a lateral Hemisection (Hx) at the second cervical (C2) spinal level. This injury model provides a historical example of respiratory plasticity: the crossed-phrenic phenomenon [CPP (Porter, 1895)]. Although this injury paralyzes the ipsilateral hemidiaphragm immediately, Porter demonstrated that transection of the contralateral phrenic nerve (paralyzing both hemidiaphragms) activated bulbospinal axons that crossed the spinal midline (decussated) below the C2 level to innervate the phrenic motor pool [reviewed in Goshgarian (2003)]. Several lines of research support this, demonstrating that CPP can be elicited soon after injury (O’Hara and Goshgarian, 1991; Goshgarian, 2003; Golder and Mitchell, 2005; Vinit et al., 2006), which suggests that this acute response does not require an anatomical change. Cross correlational analyses of phrenic nerve recordings supported this showing that post-injury function was mediated by bulbospinal pathways (Sandhu et al., 2009). However, these recordings also suggested a progressive recruitment of spinal interneurons into the injured phrenic network, which may be further contributing to functional plasticity. There is evidence of other supraspinal plasticity from sprouting monosynaptic respiratory bulbospinal projections (Vinit and Kastner, 2009; Ghali, 2017) and serotonergic centers (Bach and Mitchell, 1996; Ling et al., 2001; Zhou et al., 2001a; Hodges and Richerson, 2010; Hsu and Lee, 2015) onto phrenic circuitry.

While the focus of these anatomical studies has been on neural pathways within the spinal cord, respiratory plasticity occurs throughout the neural axis. Respiratory neuroplasticity extends throughout the CNS within the brain, brainstem, spinal cord, peripheral nervous system (Mantilla and Sieck, 2009; Nicaise et al., 2012b), spinal afferents (Iscoe and Polosa, 1976; Potts et al., 2005; Vinit et al., 2007; Nair et al., 2017), and muscle (Raineteau and Schwab, 2001; Oza and Giszter, 2014, 2015). Identifying and enhancing this anatomical plasticity is crucial to improving respiratory recovery after SCI. Another consideration is that not all plasticity is beneficial. Certainly, depending on the condition, anatomical changes can occur that worsen the potential for recovery. An important example of this in respiratory networks after human SCI is the progressive decline in respiratory muscle anatomy and function with assisted-ventilation (Powers et al., 2002, 2013; Levine et al., 2008; Smuder et al., 2016). To promote respiratory recovery post-SCI, treatments need to take these changes into account.

### Molecular Neuroplasticity

Molecular neuroplasticity encompasses an altered synthesis of cytokines, such as trophic factors, that can create a plasticity-promoting environment, attracting axons to the appropriate targets. An example is an increase in brain-derived neurotrophic factor (BDNF) and nerve growth factor (NGF) after injury or therapeutic intervention (Baker-Herman et al., 2004). Within the respiratory circuit, BDNF upregulation occurs within the phrenic motor neuron pool and is integral in enhancing anatomical plasticity (Baker-Herman et al., 2004; Sieck and Mantilla, 2009; Wilkerson and Mitchell, 2009; Mantilla et al., 2013, 2014; Gill et al., 2016; Hernandez-Torres et al., 2016; Martinez-Galvez et al., 2016) and promoting rhythmic diaphragm activity (Mantilla et al., 2013; Gransee et al., 2015).

### Functional Neural Plasticity

Functional neural plasticity is the restoration of activity in damaged pathways or increased activity in spared pathways to compensate for damage, which can occur after mild, moderate, and severe SCI (Baussart et al., 2006; Golder et al., 2011; Lane et al., 2012; Nicaise et al., 2012a,b, 2013; Awad et al., 2013; Alvarez-Argote et al., 2016). It can also occur at either the neural or behavioral level, resulting in restorative or compensatory motor function (see Box 1).

An example of *restorative plasticity* within the neural network is the CPP following a C2Hx, and restorative function within the ipsilateral diaphragm. This plasticity is characterized by the activation of ordinarily latent, contralateral respiratory bulbospinal pathways that cross the spinal midline below the injury. This restoration in function occurs after inducing a respiratory stress such as asphyxia, hypoxia, hypercapnia or contralateral phrenicotomy (Porter, 1895; Lewis and Brookhart, 1951; Goshgarian, 2003; Golder and Mitchell, 2005). In contrast, *neural compensation* is an altered (e.g., elevated) activity within non-injured respiratory circuits and respective muscles. This form of adaptive functional compensates for deficits post injury. For example, an increase of activity within the contralateral phrenic circuit after a C2Hx or C3/4 contusion injury compensates for deficits within ipsilateral circuitry (Golder et al., 2001, 2003).

*Behavioral restoration* of function is the ability to breathe in the same way after an injury as pre-injury. This ventilation can be measured through plethysmography to record the flow and tidal volume of breathing. An example of this is that following a cervical contusion injury, there is a progressive recovery toward a more normal breathing behavior in post-injury weeks (Choi et al., 2005). In contrast, *behavioral compensation* manifests as an altered pattern of ventilation after injury. An example of this is rapid, shallow breathing seen after SCI (Choi et al., 2005; Fuller et al., 2009; Golder et al., 2011; Nicaise et al., 2013; Jensen et al., 2019). This phenomenon is also seen following injuries in humans (Ledsome and Sharp, 1981; Haas et al., 1985). This change in breathing behavior likely compensates for respiratory deficits.

The extent of functional neuroplasticity and motor recovery is closely tied to anatomical plasticity and changes within the circuit or the extent of the lesion. For example, with a more mild contusive injury, there will be a higher likelihood of recovery and limited functional deficit (Alvarez-Argote et al., 2016). Restorative functional plasticity relies on anatomical pathways to be connected, or in some cases, strengthen connections, form new connections, or establish novel pathways. Accordingly, this has been reported several weeks to months following injury. In contrast, compensatory plasticity typically occurs soon after injury and initially relies solely on existing anatomical substrates. With continued change in activity within those pathways, however, there can be progressive anatomical changes that further contribute to, or reinforce, compensatory functions.

## Methods to Enhance Plasticity

**FIGURE 2:**
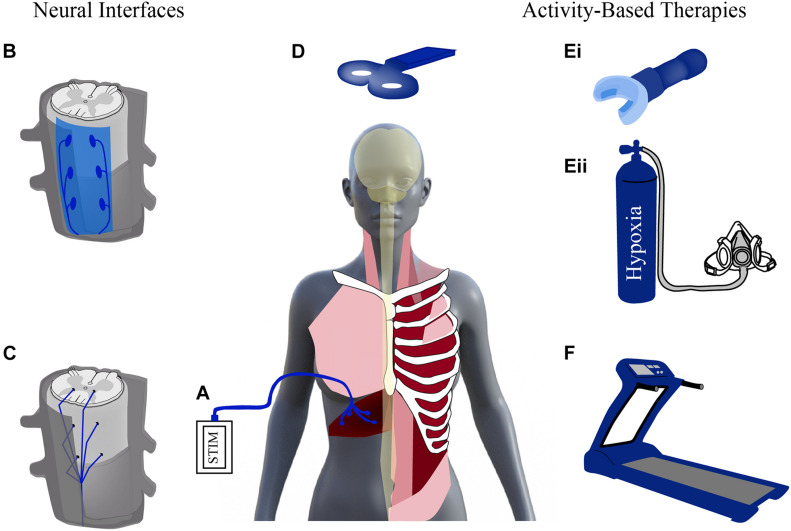
Stimulating activity to enhance plasticity. Schematic diagram of neural interfaces (left) and activity based (right) strategies to drive neural activity. Neural interfaces include strategies that can be applied to the periphery [e.g., functional electrical stimulation of diaphragm via diaphragm pacer, **(A)**]. Electrical stimulation can be applied to the spinal cord via epidural stimulation **(B)** and intraspinal stimulation **(C)** as well as non-invasively, supraspinally, via transcranial magnetic stimulation **(D)**. Activity-based therapies use physical stimuli to result in increased respiratory drive and neural stimulation via strategies like resistance training **(Ei)** or intermittent hypoxia **(Eii)**, and locomotor training, e.g., via treadmill **(F)**. Modified from Hormigo et al. (2017), Zholudeva et al. (2018).

Given the promise seen with neuroplasticity after SCI, there has been increased effort in the past decade to develop treatments capable of enhancing this plasticity and promoting respiratory recovery after injury. These treatments stimulate the nervous system either through neural interfacing (e.g., electrical stimulation) or through physical stimuli (e.g., locomotor training and respiratory rehabilitation) (Figure 2). Stimulation activates spared neural networks and can encourage the formation of new pathways, contributing to modest repair of damaged circuitry. These activation strategies can promote beneficial changes in anatomical and functional plasticity and contribute to improved outcomes after SCI. Important considerations for any of these methods will be timing and dose of the treatment, as well as, efforts to preserve adaptive plasticity and limit maladaptive plasticity.

Rapid advances are being made in both neural interfacing and electrical stimulation strategies (e.g., intraspinal, epidural, transmagnetic, and functional electrical stimulation; Figure 2). Multidisciplinary collaborations between mechanical and electrical engineers, material scientists and neurobiologists, have led to the development of highly novel and translationally appropriate devices that are being tested in both pre-clinical and clinical studies. Scientists and clinical professionals widely agree, however, that non-invasive rehabilitative strategies will always represent an effective means of helping injured individuals regain some functional improvement. Rehabilitative strategies provide the physical stimulus to enhance plasticity and provide a less invasive alternative to electrical stimulation. One example of these rehabilitative strategies is activity-based training (ABTs).

### Activity-Based Therapies

Activity-based therapies (ABTs) have extensively shown to promote neuroplasticity and improve function post-SCI in several sensory, motor, and neurological disorders (stroke, brain injury, and SCI) (Vinit et al., 2009; Dale-Nagle et al., 2010a; Hormigo et al., 2017). ABTs increase activity, often in a repeated, intermittent or “set”-like fashion, in mature neural pathways. Experimental and clinical studies have demonstrated that these ABT strategies can strengthen existing neuronal networks, stimulate synaptic and dendritic growth/plasticity, and increase baseline neuronal activity (facilitation/potentiation) (Harkema, 2001; Dunlop, 2008; Lynskey et al., 2008; Dale-Nagle et al., 2010a; Singh et al., 2011a,b; Houle and Cote, 2013; Martinez et al., 2013; Hormigo et al., 2017). These changes can also refine and prune synaptic connections and promote the recruitment of other neurons (e.g., spinal interneurons) into the neural network (Rank et al., 2015; Sandhu et al., 2015; Streeter et al., 2017). Spinal interneurons (SpINs) are a vital component of neuroplasticity (Zholudeva and Lane, 2018; Zholudeva et al., 2021), that can change their pattern of activity and are reported to alter their connectivity to contribute to novel anatomical pathways. Most importantly, this neuroplastic potential can be therapeutically driven by either electrical stimulation or ABTs (Harkema, 2008; van den Brand et al., 2012; Houle and Cote, 2013).

In an effort to better understand the mechanisms underlying therapeutically driven plasticity, several pre-clinical studies investigated changes in cytokine expression within the networks being targeted. ABTs have been shown to increase the expression of several neurotrophic factors within the injured spinal cord (Baker-Herman et al., 2004; Dunlop, 2008; Wilkerson and Mitchell, 2009). A caveat in interpreting the role of these growth factors is their widespread distribution throughout the neural axis. For, example, ABT increases BDNF expression across multiple spinal levels. Despite this, ABT-driven expression of neurotrophic factors within denervated neuronal networks may provide a non-invasive means of attracting axonal growth and enhance functional connectivity (Baker-Herman et al., 2004; Lu et al., 2005; Sieck and Mantilla, 2009; Bonner et al., 2010, 2011; Weishaupt et al., 2012, 2013; Mantilla et al., 2013; Hernandez-Torres et al., 2017). Serotonergic neurons appear to be especially responsive to increased growth factor expression. Consistent with this notion, there is increased serotonergic input onto spinal motor circuitry and increased serotonergic receptor expression (Houle and Cote, 2013). These neuroplastic molecular changes can be harnessed for therapeutic gain. As the contribution of cytokines to neuroplasticity is more clearly defined, treatments may be better refined to optimize outcome.

Perhaps the most extensively studied ABT is locomotor training, either over-ground, treadmill, or with robotics (e.g., Lokomat^®^). Locomotor training has demonstrated beneficial effects on plasticity and locomotor function following a range of SCIs, with different spinal levels and severities (Singh et al., 2011a,b; Galea et al., 2013; Hajela et al., 2013; Hillen et al., 2013; Hubli and Dietz, 2013; Martinez et al., 2013; Morawietz and Moffat, 2013; Bonizzato and Martinez, 2021). Locomotor training uses repetition to strengthen muscles, stimulate afferent feedback, enhance motor output, and thus drive related neural plasticity (Harkema, 2001).

While historically the focus of locomotor training was to improve locomotion, it has also been shown to improve a range of non-locomotor functions, including bladder (Ward et al., 2014) and cardiovascular function (Ditor et al., 2005a,b; Hicks and Ginis, 2008). More recent studies have also demonstrated that treadmill training can enhance respiratory recovery in people with chronic cervical and thoracic injuries (Terson de Paleville et al., 2013). This improvement in respiratory function was speculated to be due to increased heart rate and minute ventilation (increase cardiopulmonary activity) during treadmill training (Terson de Paleville et al., 2013). However, the extent of respiratory improvement may also be “dose-dependent.” Terson de Paleville saw improvements in respiratory function for subjects who received 60 min of stepping on a treadmill, 5 days a week for an average of about 12 weeks (Terson de Paleville et al., 2013). In contrast, individuals who received passive robot-assisted stepping did not improve cardiopulmonary function (Jack et al., 2011). One limitation might be achieving sufficient increase in limb afferent stimulation to encourage locomotor-respiratory coupling post-SCI (Sherman et al., 2009). This hypothesis is supported by hindlimb stimulation (a passive event) producing respiratory rhythm entrainment (Iscoe and Polosa, 1976; Morin and Viala, 2002; Potts et al., 2005), increasing phrenic motor output (Persegol et al., 1993).

While the mechanisms explaining how locomotor training can promote respiratory plasticity remain unclear, there are some lines of evidence suggesting that training in the activity you wish to recover might provide a more direct and efficacious strategy. Thus, there has been growing interest in the field of SCI, to entrain respiratory plasticity by stimulating and increasing respiratory activity.

### Respiratory Training

**FIGURE 3:**
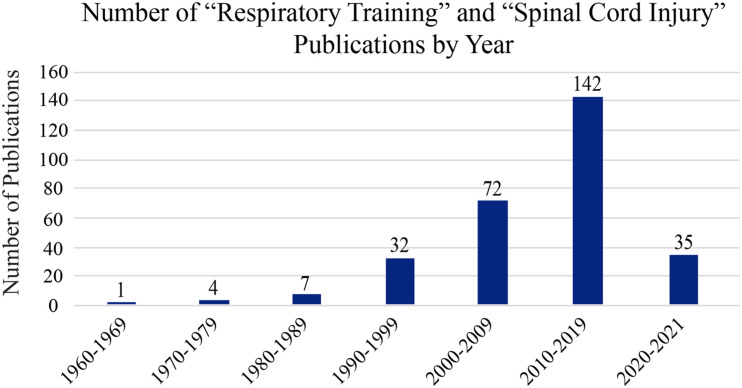
Publication history of respiratory training post-SCI. A PubMed search was made using the terms “respiratory training” and “spinal cord injury” to assess the relative number of research papers on this topic over the last 60 years. As the image shows, interest on this topic is increasing exponentially with each decade.

Respiratory training is the repetitive activation (either electrical or physical) of inspiratory and expiratory muscles in a systematic way to strengthen respiratory pathways and the muscles they innervate. The term “respiratory training” originated from respiratory axillary muscle training to improve breathing after cervical SCI in 1967 (Imamura, 1967). As the number of publications on respiratory training and SCI continues to increase, so has the definition and use for “respiratory training” (Figure 3). While the origins of respiratory training are within exercise physiology, it has also been used in elderly populations and for many disorders such as chronic obstructive pulmonary disease (COPD), Parkinson’s disease, multiple sclerosis, speech pathologies, and voice disorders (Sapienza and Wheeler, 2006). Respiratory training now broadly refers to strengthening the primary and accessory (including inspiratory and expiratory) respiration muscles (Sapienza and Wheeler, 2006; Sapienza et al., 2011). These are further divided into inspiratory and expiratory training strategies (Bolser et al., 2009; Martin et al., 2011; Sapienza et al., 2011; Laciuga et al., 2014). Deciding which training paradigm to use depends on the needs of the individual. For example, an individual with a high cervical injury will have inspiratory and expiratory deficits and will require a training technique that targets both muscle groups. However, an individual with a lower thoracic injury may require techniques targeting expiratory muscles.

### Resistance Muscle Strength Training

The goal of any *muscle strength training (MST)* is to enhance the ability of the neuromuscular system to respond to a demand of gradually increasing intensity. This intensity is defined in terms of load amount and duration of the exercise task (e.g., minutes per day × days per week × total weeks) (Sapienza and Wheeler, 2006). The total stimulus should increase the activity of the neuromuscular system beyond the normal level (Mueller et al., 2006) and drive it to adapt to increased demand (Sapienza and Wheeler, 2006). Typical MST paradigms in the clinic consist of three sessions (with 25–30 repetitions), 3–5 days per week, 4–8 weeks (Carpinelli and Otto, 1998; Schlumberger et al., 2001; Rhea et al., 2002; Sapienza et al., 2011). The intensity of MST can directly affect improvement in respiratory muscle strength (Raab et al., 2019).

There are two main MST strategies: resistance and threshold training. Resistance MST consists of breathing through a small diameter hole, making the participant breathe harder due to the limited airflow (Sapienza and Wheeler, 2006; Berlowitz and Tamplin, 2013; Raab et al., 2019). It can be targeted toward either inspiratory or expiratory muscles (Roth et al., 2010) or combined (Kim et al., 2017). Combined training resulted in increased forced vital capacity and expiratory volume, demonstrating improved pulmonary function compared to the respiratory muscle training alone and control group (Kim et al., 2017). Threshold MST forces the individual to modulate their breathing to overcome a spring-loaded valve controlling the airflow (Sapienza and Wheeler, 2006; Galeiras Vazquez et al., 2013; Raab et al., 2019). Resistance and threshold MST result in improved breathing, facilitates weaning from mechanical ventilation (Aldrich et al., 1989; Martin et al., 2011; Smith et al., 2014), and has beneficial effects in secondary respiratory behaviors [e.g., sneezing, sniffing, or coughing (Postma et al., 2015; Aslan et al., 2016; Legg Ditterline et al., 2018; Shin et al., 2019)].

### Altering Inhaled Air for Respiratory Training

An alternative to direct electrical stimulation or resistance training of respiratory muscles is non-invasive peripheral and central chemoreceptor activation. For example, hypoxia (decreased oxygen) and hypercapnia (increased carbon dioxide) have been used to elicit activity within the phrenic network (Millhorn et al., 1980; Nielsen et al., 1986). These types of chemical activation have been used to stimulate respiratory drive and elicit neuroplasticity non-invasively. For example, Millhorn et al. (1980) discovered that stimulation of the peripheral and central chemoreceptors resulted in a lasting increase of phrenic activity (Millhorn et al., 1980). Building on this Bach and Mitchell (1996) used three, 5-min bursts of hypoxia (intermittent with room air) to stimulate this chemoreceptor activity and elicit a lasting (hours) increase in phrenic nerve activity (Bach and Mitchell, 1996), termed long-term phrenic facilitation (LTF). LTF is an example of respiratory neuroplasticity characterized by a period of enhanced neural output following a single stimulation paradigm (Fuller et al., 2000; Mitchell et al., 2001). When the same paradigm was applied to hypercapnia (10% CO_2_) stimulation paradigm resulted in long-term depression (LTD), effectively decreasing phrenic nerve output (Bach and Mitchell, 1996, 1998). Important to note is lowering CO_2_ levels (to 5%) or limiting exposure to 3–5 min does not elicit this LTD (Baker and Mitchell, 2000; Baker et al., 2001). These episodic exposures also elicit LTF for hypoxia and hypercapnia, but not continuous exposure paradigms (Baker and Mitchell, 2000; Baker et al., 2001).

Increased phrenic plasticity from intermittent hypoxia or hypercapnia led to using these strategies as an alternative method of “respiratory training.” This form of respiratory training is modeled after other rehabilitative ABTs [reviewed in Dale-Nagle et al. (2010b); Dale et al. (2014), Gonzalez-Rothi et al. (2015, 2021)]. Most importantly, this training activates chemoreceptors to drive respiration and provides a non-invasive means of attracting axonal growth, enhancing respiratory functional connectivity to improve breathing (Baker-Herman et al., 2004; Lu et al., 2005; Sieck and Mantilla, 2009; Bonner et al., 2010, 2011; Weishaupt et al., 2012, 2013; Mantilla et al., 2013; Hernandez-Torres et al., 2017).

#### Intermittent Hypoxia

Intermittent hypoxia (IH) has been studied both experimentally and clinically as a non-invasive means of stimulating respiratory output. This “activity-based” respiratory training has been used to enhance neuroplasticity, particularly with a focus on the phrenic network, and, improved respiration (Fuller et al., 2003; Mitchell and Johnson, 2003; Vinit et al., 2009; Wilkerson and Mitchell, 2009). While a vast range of paradigms have been developed to test IH, the three most commonly reported strategies used in rodent models are:

• Acute intermittent hypoxia (AIH); short exposures (e.g., 3 × 5 min each, or 5 × 3 min each), given in a single day.
• Daily acute intermittent hypoxia (dAIH; short, daily exposures over several days (e.g., 10 hypoxia episodes per day for 5–7 days.
• Chronic intermittent hypoxia (CIH); e.g., 72 episodes of hypoxia for 1–2 weeks or more.

Examples of these studies are reviewed in Dale-Nagle et al. (2010b). All paradigms effectively improve respiratory outcomes at multiple time points, including 2–10 weeks post spinal cord injury in rodents (Ling et al., 2001; Dale-Nagle et al., 2010b). While chronic intermittent hypoxia was able to enhance plasticity at the level of the phrenic motor pool and enhance crossed phrenic pathways (Fuller et al., 2003), it also led to significant cognitive (Row, 2007), metabolic (Tasali and Ip, 2008), and hypertensive (Fletcher et al., 1992) deficits, and decreased levels of BDNF within the hippocampus (Vinit et al., 2009; Xie et al., 2010; Navarrete-Opazo and Mitchell, 2014). Therefore, almost all IH training paradigms are now done daily with acute intermittent timing (Dale-Nagle et al., 2010b; Gonzalez-Rothi et al., 2015).

**FIGURE 4:**
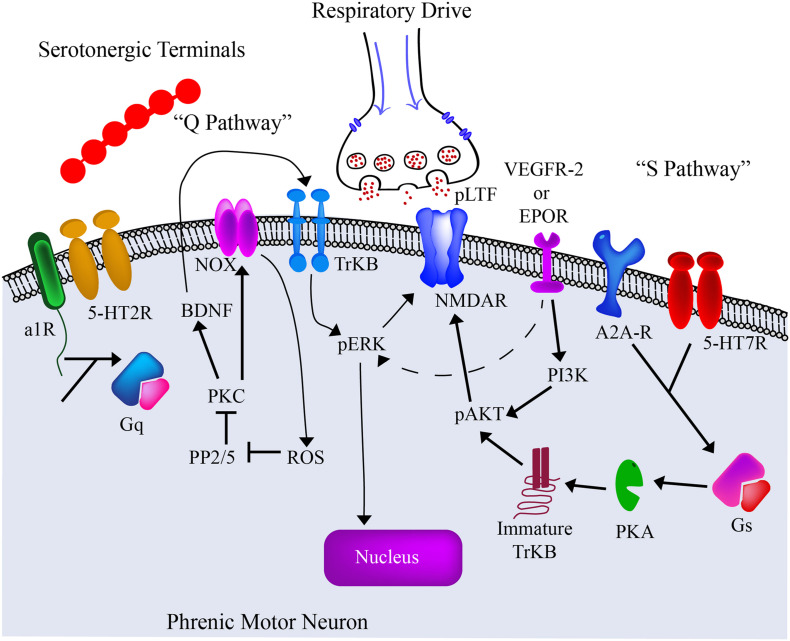
The current model of the “Q” and “S” Pathways. The past several decades has seen significant advances in our understanding of intermittent hypoxia and its effects on the spinal phrenic network. Particular focus has been given to changes on the phrenic motoneuron itself. From these ongoing studies we are gaining an appreciation of the cellular receptors and intracellular pathways that contribute to plasticity and altered motor function under different respiratory conditions. The current pre-clinical and clinical goal is to employ therapeutic strategies that can harness these mechanisms and enhance motor output after spinal cord injury or disease. Adapted from Dale-Nagle et al. (2010b); Gonzalez-Rothi et al. (2015).

The mechanisms by which hypoxia induces LTF and phrenic plasticity are both complex and multifaceted. IH respiratory training has demonstrated the ability to elicit serotonin dependent plasticity (Ling et al., 2001; Mitchell et al., 2001; Baker-Herman and Mitchell, 2002; Golder and Mitchell, 2005; Dale-Nagle et al., 2010b; Devinney et al., 2013), and enhance bulbospinal axon sprouting into phrenic circuitry (Baker-Herman et al., 2004; Dale-Nagle et al., 2010b; Gonzalez-Rothi et al., 2015). There are two main pathways described as the “Q” and “S” pathways of promoting neuroplasticity [reviewed in Dale et al. (2014); Hassan et al. (2018); Figure 4]. These pathways get their name from the primary type of G protein-coupled receptor (Gs or Gq) activated.

**FIGURE 5:**
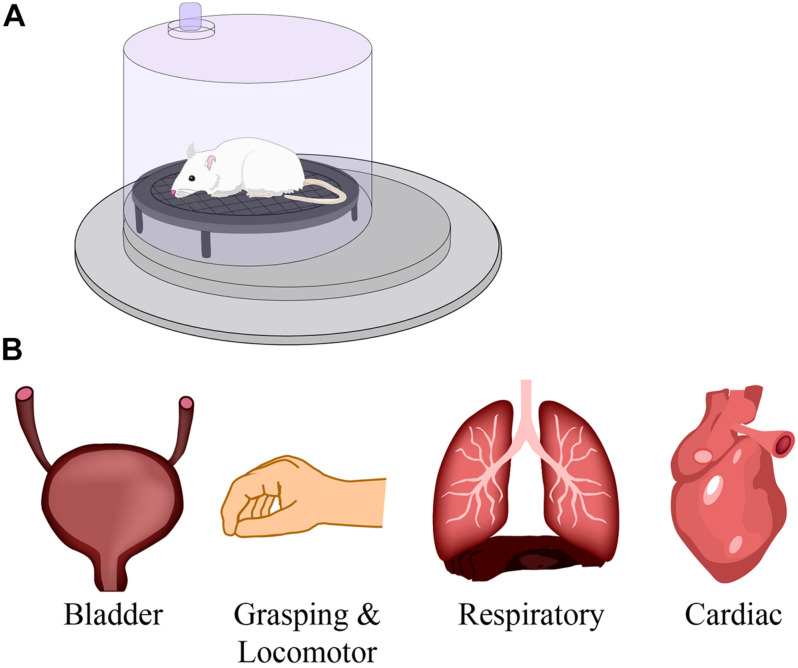
The effect of respiratory training on neural networks. The studies described in this review use respiratory training, such as intermittent hypoxia and hypercapnia in a pre-clinical model **(A)**, to improve respiratory function after spinal cord injury. However, numerous studies have reported beneficial effects on other motor networks such as bladder, grasping and locomotion, and cardiac systems **(B)**. Modified from Zholudeva and Lane (2019).

In addition to upregulating molecular markers for plasticity, hypoxia has also been shown to enhance interneuronal plasticity and connectivity, and alter motor output. Studies have identified that spinal interneurons (SpINs) can respond to hypoxia (Lane et al., 2009; Sandhu et al., 2015) and can be recruited following IH training (Streeter et al., 2017).

Intermittent hypoxia training has also been shown to increase plasticity in non-respiratory networks (Supplementary Table 1 and Figure 5). Pre-clinical studies reported 7 days of IH in rats with cervical SCI improved performance on the horizontal ladder test (Lovett-Barr et al., 2012; Prosser-Loose et al., 2015; Hassan et al., 2018). IH has also been used in conjunction with specific tasks resulting in synergistic improvements in locomotion (Lovett-Barr et al., 2012), reaching and grasping techniques (Prosser-Loose et al., 2015).

Building on the pre-clinical data, clinical studies first focused on ankle flexion in chronic incomplete SCI individuals (see Supplementary Table 2). IH training significantly improved maximal plantarflexion torque and gastrocnemius electromyographic activity that lasted up to 4 h after the initial IH administration (Trumbower et al., 2012). This not only demonstrated a persistent neuroplastic effect of IH training, but provided evidence of enhanced motor function in people living with SCI. IH training was subsequently shown to improve both walking speed 10-Meter Walk Test (10MWT), distance and endurance 6-Minute Walk Test (6MWT) at 1 day and 1 week during training, and the 1 week follow up (Hayes et al., 2014). Combined IH training with 30 min of overground walking, showed even greater improvement in locomotion speed and distance (Hayes et al., 2014). This improvement may demonstrate that combinatorial therapies may promote greater synergistic functional benefits in injured individuals (Hayes et al., 2014). More recent use of IH training has shown that there is a persistent effect in locomotor facilitation over time and that this can be further maintained with three IH treatments per week after the initial combinatorial walk/IH training (Navarrete-Opazo et al., 2017a). Similarly, combined IH training with hand exercises revealed improved hand dexterity function and maximum hand opening in all participants (Trumbower et al., 2017).

Compromised bladder and bowel function has profound impacts on quality of life for those living with SCI, including a loss of independence, increased risk of infection from catheter use or from incomplete bladder voiding, and autonomic dysreflexia. While there are no clinical studies looking at IH and bladder and bowel function, some animal models are investigating IH and lower urinary tract plasticity. In brief, Collins et al. (2017) revealed that IH-induced neuroplasticity can improve lower urinary tract function in rats with chronic incomplete SCI and may provide a non-invasive method of improving bladder function within the SCI patient population (Collins et al., 2017).

Another respiratory deficit that arises following cervical SCI is sleep-disordered breathing. A consequence of this is obstructive sleep apnea that can result in chronic episodes of hypoxia and hypercapnia, contributing to cardiovascular morbidity, high blood pressure, increased sympathetic nerve activity, cardiac arrhythmia and myocardial infarction (Prabhakar et al., 2005). However, IH consisting of 3–4 rounds of 5–7 min exposures at 12–10% O_2_ for 2–3 weeks can benefit cardiovascular diseases such as decreased hypertension, coronary heart disease, and heart failure (Serebrovskaya and Xi, 2016). While these initial studies were conducted on spinally intact individuals, future work can begin to assess the potential in people living with SCI.

In summary, IH has demonstrated the ability to improve respiratory function, elicit serotonin and neurotrophic factor dependent plasticity, enhance bulbospinal axonal sprouting into active phrenic circuitry, and recruit populations of SpINs. Clinically, IH training has also been investigated for its ability to promote recovery of both respiratory (Vinit et al., 2009; Tester et al., 2014) and non-respiratory (Trumbower et al., 2012, 2017; Dale et al., 2014; Hayes et al., 2014) motor functions.

#### Intermittent Hypercapnia

Like hypoxia, exposure to hypercapnia (elevated CO_2_) has also been used to increase respiratory drive via central and peripheral chemoreceptor activation. There is evidence that hypercapnia and hypoxia activate chemoreceptors differently (Long et al., 1994) and that hypercapnia can act as a stronger respiratory stimulant than hypoxia (Somers et al., 1989; Nattie and Li, 2012). This chemoreceptor activity is also enhanced in individuals with chronic SCI compared to non-injured individuals (Bascom et al., 2016).

Hypercapnia as a respiratory stimulus has been shown to increase activity within several brainstem nuclei, including the retrotrapezoid nucleus (RTN) and those within the ventral respiratory column (VRC) (Millhorn and Eldridge, 1986; Guyenet et al., 2012, 2019; Molkov et al., 2014; Wakai et al., 2015). Following hypercapnia exposure, there is an increased drive from the RTN to the VRC resulting in increased amplitude and frequency of phrenic output (Molkov et al., 2014). Within the nucleus tractus solitarius, the principal visceral sensory nucleus, PHOX2B-expressing neurons exhibit CO_2_ sensitivity and increase activity after exposure to hypercapnia (Fu et al., 2019). Another crucial effect of hypercapnia on brainstem nuclei is the activation of the dorsal raphe (containing serotonergic neurons) (Smith et al., 2018; Kaur et al., 2020). Because carotid chemoafferents also activate raphe, there is also reason to believe that exposure to hypercapnia and hypoxia may further enhance serotonin-dependent mechanisms of plasticity beyond hypoxia alone (Welch, 2021).

While plasticity pathways are well studied following IH, the molecular changes post hypercapnia are not well defined. Overall, hypercapnia is known to upregulate many transcription factors responsible for respiration, motor, and immune function [reviewed in Shigemura et al. (2020)]. In light of the documented “S” and “Q” Pathways (Figure 4), hypercapnia is believed to activate the A2a receptors (Bach and Mitchell, 1998; Kinkead et al., 2001) as part of the initial “S” pathway. Consistent with this, exposure to severe hypercapnia (10% CO_2_) inhibits plasticity, resulting in long-term phrenic depression (LTD), which is attenuated with the delivery of an A2a receptor antagonist (Bach and Mitchell, 1998). However, it is important to note that lower hypercapnia concentrations (3–5% CO_2_) does not elicit LTD (Bach and Mitchell, 1998), and thus may drive other molecular pathways.

While hypoxia has been shown to have has a greater effect on respiratory timing, hypercapnia has a more significant effect on peak phrenic nerve activity (Ledlie et al., 1981). Together hypoxia and hypercapnia exposure demonstrate excitation to increase muscle sympathetic nerve activity (Jouett et al., 2015). Also, combined hypoxia and hypercapnia exposure leads to an increase in ipsilateral diaphragm activity but not intercostal activity after a mid-cervical contusion (Wen and Lee, 2018). Furthermore, intermittent hypoxia-hypercapnia following mid-cervical contusion induces an increase in tidal volume, whereas inactivation of the 5-HT7 receptor (Gs coupled protein) combined with this treatment further transiently improved this recovery (Wu et al., 2020). However, more studies need to be done to further understand the implication of the Gs or Gq pathway in this recovery.

A potential therapeutic advantage of hypercapnia training is that unlike IH it maintains normoxia. It has also been shown that hypercapnia can act as a more potent respiratory stimulus than hypoxia (Somers et al., 1989; Nattie and Li, 2012). Increased respiratory neural drive (brainstem) results in increased phrenic output (phrenic nerve and diaphragm) which contributes to entrainment of spared circuits after SCI, activation of latent pathways (Zhou et al., 2001b; Zimmer et al., 2007), as well as anatomical plasticity (e.g., the formation of novel neural circuits) (Baker et al., 2001; Feldman et al., 2003). Apart from anatomical plasticity, intermittent hypercapnia elicits functional changes in respiratory circuits after SCI (Baker et al., 2001). A summary of studies using hypercapnia to enhance anatomical and functional neural plasticity is provided in Supplementary Table 3.

## Closing Remarks

With the mounting clinical and experimental evidence for plasticity after spinal cord injury, tremendous effort is being made to develop treatments that can reduce maladaptive changes, and act synergistically with ongoing adaptive changes, to further optimize the benefits of neuroplasticity. These neural interfacing and activity-based therapies are being extensively clinically tested, which also speaks to their translational relevance. Combining neural interfacing with activity-based therapies has already shown to be effective for promoting recovery of non-respiratory functions (van den Brand et al., 2012), so it is tempting to predict that similar benefits may be achievable for respiratory functions. Even greater benefit may come from combining these approaches with other therapies, such as cellular or biomaterial transplantation, or administration of pro-regenerative compounds, that can promote greater anatomical growth and repair. The future of therapeutic development for respiratory function and plasticity after spinal cord injury holds great promise.

## Author Contributions

All authors listed have made a substantial, direct and intellectual contribution to the work, and approved it for publication.

## Conflict of Interest

The authors declare that the research was conducted in the absence of any commercial or financial relationships that could be construed as a potential conflict of interest.

## Publisher’s Note

All claims expressed in this article are solely those of the authors and do not necessarily represent those of their affiliated organizations, or those of the publisher, the editors and the reviewers. Any product that may be evaluated in this article, or claim that may be made by its manufacturer, is not guaranteed or endorsed by the publisher.

## References

[B1] Aldrich T. K. Karpel J. P. Uhrlass R. M. Sparapani M. A. Eramo D. Ferranti R. (1989). Weaning from mechanical ventilation: adjunctive use of inspiratory muscle resistive training. *Crit. Care Med.* 17 143–147. 10.1097/00003246-198902000-00008 2914446

[B2] Alvarez-Argote S. Gransee H. M. Mora J. C. Stowe J. M. Jorgenson A. J. Sieck G. C. (2016). The Impact of Midcervical Contusion Injury on Diaphragm Muscle Function. *J. Neurotrauma* 33 500–509. 26413840 10.1089/neu.2015.4054 PMC4779319

[B3] Anke A. Aksnes A. K. Stanghelle J. K. Hjeltnes N. (1993). Lung volumes in tetraplegic patients according to cervical spinal cord injury level. *Scand. J. Rehabil. Med.* 25 73–77. 8341994

[B4] Arnold B. M. Toosi B. M. Caine S. Mitchell G. S. Muir G. D. (2021). Prolonged acute intermittent hypoxia improves forelimb reach-to-grasp function in a rat model of chronic cervical spinal cord injury. *Exp. Neurol.* 340:113672. 10.1016/j.expneurol.2021.113672 33652030

[B5] Aslan S. C. Randall D. C. Krassioukov A. V. Phillips A. Ovechkin A. V. (2016). Respiratory Training Improves Blood Pressure Regulation in Individuals With Chronic Spinal Cord Injury. *Arch. Phys. Med. Rehabil.* 97 964–973. 26718236 10.1016/j.apmr.2015.11.018 PMC4884550

[B6] Awad B. I. Warren P. M. Steinmetz M. P. Alilain W. J. (2013). The role of the crossed phrenic pathway after cervical contusion injury and a new model to evaluate therapeutic interventions. *Exp. Neurol.* 248 398–405. 23886671 10.1016/j.expneurol.2013.07.009

[B7] Bach K. B. Mitchell G. S. (1996). Hypoxia-induced long-term facilitation of respiratory activity is serotonin dependent. *Respir. Physiol.* 104 251–260. 10.1016/0034-5687(96)00017-5 8893371

[B8] Bach K. B. Mitchell G. S. (1998). Hypercapnia-induced long-term depression of respiratory activity requires alpha2-adrenergic receptors. *J. Appl. Physiol.* 84 2099–2105. 9609805 10.1152/jappl.1998.84.6.2099

[B9] Baker T. L. Fuller D. D. Zabka A. G. Mitchell G. S. (2001). Respiratory plasticity: differential actions of continuous and episodic hypoxia and hypercapnia. *Respir. Physiol.* 129 25–35. 11738644 10.1016/s0034-5687(01)00280-8

[B10] Baker T. L. Mitchell G. S. (2000). Episodic but not continuous hypoxia elicits long-term facilitation of phrenic motor output in rats. *J. Physiol.* 529 215–219. 11080263 10.1111/j.1469-7793.2000.00215.x PMC2270180

[B11] Baker-Herman T. L. Fuller D. D. Bavis R. W. Zabka A. G. Golder F. J. Doperalski N. J. (2004). BDNF is necessary and sufficient for spinal respiratory plasticity following intermittent hypoxia. *Nat. Neurosci.* 7 48–55. 14699417 10.1038/nn1166

[B12] Baker-Herman T. L. Mitchell G. S. (2002). Phrenic long-term facilitation requires spinal serotonin receptor activation and protein synthesis. *J. Neurosci.* 22 6239–6246. 12122082 10.1523/JNEUROSCI.22-14-06239.2002 PMC6757927

[B13] Bascom A. T. Sankari A. Badr M. S. (2016). Spinal cord injury is associated with enhanced peripheral chemoreflex sensitivity. *Physiol. Rep.* 4:e12948. 10.14814/phy2.12948 PMC5027355 27597767

[B14] Baussart B. Stamegna J. C. Polentes J. Tadie M. Gauthier P. (2006). A new model of upper cervical spinal contusion inducing a persistent unilateral diaphragmatic deficit in the adult rat. *Neurobiol. Dis.* 22 562–574. 16488616 10.1016/j.nbd.2005.12.019

[B15] Berlowitz D. J. Tamplin J. (2013). Respiratory muscle training for cervical spinal cord injury. *Cochrane Database Syst. Rev.* 7:CD008507. 10.1002/14651858.CD008507.pub2 23881660 PMC11089516

[B16] Bolser D. C. Jefferson S. C. Rose M. J. Tester N. J. Reier P. J. Fuller D. D. (2009). Recovery of airway protective behaviors after spinal cord injury. *Respir. Physiol. Neurobiol.* 169 150–156. 10.1016/j.resp.2009.07.018 19635591 PMC2789652

[B17] Bonizzato M. Martinez M. (2021). An intracortical neuroprosthesis immediately alleviates walking deficits and improves recovery of leg control after spinal cord injury. *Sci. Transl. Med.* 13:eabb4422. 10.1126/scitranslmed.abb4422 33762436

[B18] Bonner J. F. Blesch A. Neuhuber B. Fischer I. (2010). Promoting directional axon growth from neural progenitors grafted into the injured spinal cord. *J. Neurosci. Res.* 88 1182–1192. 10.1002/jnr.22288 19908250 PMC2844860

[B19] Bonner J. F. Connors T. M. Silverman W. F. Kowalski D. P. Lemay M. A. Fischer I. (2011). Grafted neural progenitors integrate and restore synaptic connectivity across the injured spinal cord. *J. Neurosci.* 31 4675–4686. 10.1523/JNEUROSCI.4130-10.2011 21430166 PMC3148661

[B20] Brown R. Dimarco A. F. Hoit J. D. Garshick E. (2006). Respiratory dysfunction and management in spinal cord injury. *Respir. Care* 51 853–868. 16867197 PMC2495152

[B21] Carpinelli R. N. Otto R. M. (1998). Strength training. Single versus multiple sets. *Sports Med* 26 73–84. 10.2165/00007256-199826020-00002 9777681

[B22] Choi H. Liao W. L. Newton K. M. Onario R. C. King A. M. Desilets F. C. (2005). Respiratory abnormalities resulting from midcervical spinal cord injury and their reversal by serotonin 1A agonists in conscious rats. *J. Neurosci.* 25 4550–4559. 10.1523/JNEUROSCI.5135-04.2005 15872102 PMC6725034

[B23] Christiansen L. Chen B. Lei Y. Urbin M. A. Richardson M. S. A. Oudega M. (2021). Acute intermittent hypoxia boosts spinal plasticity in humans with tetraplegia. *Exp. Neurol.* 335:113483. 10.1016/j.expneurol.2020.113483 32987000 PMC9208274

[B24] Ciesla M. C. Seven Y. B. Allen L. L. Smith K. N. Asa Z. A. Simon A. K. (2021). Serotonergic innervation of respiratory motor nuclei after cervical spinal injury: impact of intermittent hypoxia. *Exp. Neurol.* 338:113609. 10.1016/j.expneurol.2021.113609 33460645 PMC8327480

[B25] Collins W. Phagu N. Catege M. Solomon I. (2017). Improvement in Lower Urinary Tract Function Following a Single Bout of Acute Intermittent Hypoxia in Rats with Chronic Spinal Cord Injury. *FASEB J.* 31 724.8–724.8.

[B26] Dalal K. DiMarco A. F. (2014). Diaphragmatic pacing in spinal cord injury. *Phys. Med. Rehabil. Clin. N. Am.* 25 619–629. 10.1016/j.pmr.2014.04.004 25064791

[B27] Dale E. A. Ben Mabrouk F. Mitchell G. S. (2014). Unexpected benefits of intermittent hypoxia: enhanced respiratory and nonrespiratory motor function. *Physiology* 29 39–48. 10.1152/physiol.00012.2013 24382870 PMC4073945

[B28] Dale-Nagle E. A. Hoffman M. S. Macfarlane P. M. Mitchell G. S. (2010a). Multiple pathways to long-lasting phrenic motor facilitation. *Adv. Exp. Med. Biol.* 669 225–230. 10.1007/978-1-4419-5692-7_45 20217354 PMC3021942

[B29] Dale-Nagle E. A. Hoffman M. S. Macfarlane P. M. Satriotomo I. Lovett-Barr M. R. Vinit S. (2010b). Spinal plasticity following intermittent hypoxia: implications for spinal injury. *Ann. N. Y. Acad. Sci.* 1198 252–259. 10.1111/j.1749-6632.2010.05499.x 20536940 PMC3030965

[B30] Darlot F. Cayetanot F. Gauthier P. Matarazzo V. Kastner A. (2012). Extensive respiratory plasticity after cervical spinal cord injury in rats: axonal sprouting and rerouting of ventrolateral bulbospinal pathways. *Exp. Neurol.* 236 88–102. 10.1016/j.expneurol.2012.04.004 22542946

[B31] De Troyer A. Estenne M. (1990). Chest wall motion in paraplegic subjects. *Am. Rev. Respir. Dis.* 141 332–336. 10.1164/ajrccm/141.2.332 2137312

[B32] De Troyer A. Estenne M. Vincken W. (1986). Rib cage motion and muscle use in high tetraplegics. *Am. Rev. Respir. Dis.* 133 1115–1119. 2940952 10.1164/arrd.1986.133.6.1115

[B33] Devinney M. J. Huxtable A. G. Nichols N. L. Mitchell G. S. (2013). Hypoxia-induced phrenic long-term facilitation: emergent properties. *Ann. N. Y. Acad. Sci.* 1279 143–153. 10.1111/nyas.12085 23531012 PMC3880582

[B34] DeVivo M. J. Black K. J. Stover S. L. (1993). Causes of death during the first 12 years after spinal cord injury. *Arch. Phys. Med. Rehabil.* 74 248–254. 8439250

[B35] Diep T. T. Khan T. R. Zhang R. Duffin J. (2007). Long-term facilitation of breathing is absent after episodes of hypercapnic hypoxia in awake humans. *Respir. Physiol. Neurobiol.* 156 132–136. 10.1016/j.resp.2006.08.011 17027347

[B36] DiMarco A. F. (2005). Restoration of respiratory muscle function following spinal cord injury. Review of electrical and magnetic stimulation techniques. *Respir. Physiol. Neurobiol.* 147 273–287. 10.1016/j.resp.2005.03.007 16046197

[B37] Ditor D. S. Kamath M. V. Macdonald M. J. Bugaresti J. Mccartney N. Hicks A. L. (2005a). Effects of body weight-supported treadmill training on heart rate variability and blood pressure variability in individuals with spinal cord injury. *J. Appl. Physiol.* 98 1519–1525. 10.1152/japplphysiol.01004.2004 15563629

[B38] Ditor D. S. Macdonald M. J. Kamath M. V. Bugaresti J. Adams M. Mccartney N. (2005b). The effects of body-weight supported treadmill training on cardiovascular regulation in individuals with motor-complete SCI. *Spinal Cord* 43 664–673. 10.1038/sj.sc.3101785 15968298

[B39] Doperalski N. J. Fuller D. D. (2006). Long-term facilitation of ipsilateral but not contralateral phrenic output after cervical spinal cord hemisection. *Exp. Neurol.* 200 74–81. 10.1016/j.expneurol.2006.01.035 16647702

[B40] Dougherty B. J. Terada J. Springborn S. R. Vinit S. Macfarlane P. M. Mitchell G. S. (2018). Daily acute intermittent hypoxia improves breathing function with acute and chronic spinal injury via distinct mechanisms. *Respir. Physiol. Neurobiol.* 256 50–57. 10.1016/j.resp.2017.05.004 28549897 PMC5701887

[B41] Dunlop S. A. (2008). Activity-dependent plasticity: implications for recovery after spinal cord injury. *Trends Neurosci.* 31 410–418. 10.1016/j.tins.2008.05.004 18602172

[B42] Engwall M. J. Vidruk E. H. Nielsen A. M. Bisgard G. E. (1988). Response of the goat carotid body to acute and prolonged hypercapnia. *Respir. Physiol.* 74 335–344. 10.1016/0034-5687(88)90041-2 3222565

[B43] Feldman J. L. (1986). “Neurophysiology of breathing in mammals,” in *Handbook of Physiology - The Nervous System IV*, ed. Bloom F. E. (Bethesda: American Physiological Society), 463–524. 10.1002/cphy.cp010409

[B44] Feldman J. L. Mitchell G. S. Nattie E. E. (2003). Breathing: rhythmicity, plasticity, chemosensitivity. *Annu. Rev. Neurosci.* 26 239–266. 10.1146/annurev.neuro.26.041002.131103 12598679 PMC2811316

[B45] Fletcher E. C. Lesske J. Qian W. Miller C. C. III Unger T. (1992). Repetitive, episodic hypoxia causes diurnal elevation of blood pressure in rats. *Hypertension* 19 555–561. 10.1161/01.HYP.19.6.555 1592450

[B46] Fu C. Shi L. Wei Z. Yu H. Hao Y. Tian Y. (2019). Activation of Phox2b-Expressing Neurons in the Nucleus Tractus Solitarii Drives Breathing in Mice. *J. Neurosci.* 39 2837–2846. 10.1523/JNEUROSCI.2048-18.2018 30626698 PMC6462453

[B47] Fuller D. D. Bach K. B. Baker T. L. Kinkead R. Mitchell G. S. (2000). Long term facilitation of phrenic motor output. *Respir. Physiol.* 121 135–146. 10.1016/S0034-5687(00)00124-9 10963770

[B48] Fuller D. D. Doperalski N. J. Dougherty B. J. Sandhu M. S. Bolser D. C. Reier P. J. (2008). Modest spontaneous recovery of ventilation following chronic high cervical hemisection in rats. *Exp. Neurol.* 211 97–106. 10.1016/j.expneurol.2008.01.013 18308305 PMC2613014

[B49] Fuller D. D. Johnson S. M. Olson E. B. Jr. Mitchell G. S. (2003). Synaptic pathways to phrenic motoneurons are enhanced by chronic intermittent hypoxia after cervical spinal cord injury. *J. Neurosci.* 23 2993–3000. 10.1523/JNEUROSCI.23-07-02993.2003 12684486 PMC6742063

[B50] Fuller D. D. Sandhu M. S. Doperalski N. J. Lane M. A. White T. E. Bishop M. D. (2009). Graded unilateral cervical spinal cord injury and respiratory motor recovery. *Respir. Physiol. Neurobiol.* 165 245–253. 10.1016/j.resp.2008.12.010 19150658 PMC2646795

[B51] Galea M. P. Dunlop S. A. Davis G. M. Nunn A. Geraghty T. Hsueh Y. S. (2013). Intensive exercise program after spinal cord injury (”Full-On”): study protocol for a randomized controlled trial. *Trials* 14:291. 10.1186/1745-6215-14-291 24025260 PMC3848453

[B52] Galeiras Vazquez R. Rascado Sedes P. Mourelo Farina M. Montoto Marques A. Ferreiro Velasco M. E. (2013). Respiratory management in the patient with spinal cord injury. *Biomed. Res. Int.* 2013:168757. 10.1155/2013/168757 24089664 PMC3781830

[B53] Garshick E. Kelley A. Cohen S. A. Garrison A. Tun C. G. Gagnon D. (2005). A prospective assessment of mortality in chronic spinal cord injury. *Spinal Cord* 43 408–416. 10.1038/sj.sc.3101729 15711609 PMC1298182

[B54] Ghali M. G. (2017). The bulbospinal network controlling the phrenic motor system: laterality and course of descending projections. *Neurosci. Res.* 121 7–17. 10.1016/j.neures.2017.03.004 28389264

[B55] Gill L. C. Gransee H. M. Sieck G. C. Mantilla C. B. (2016). Functional recovery after cervical spinal cord injury: role of neurotrophin and glutamatergic signaling in phrenic motoneurons. *Respir. Physiol. Neurobiol.* 226 128–136. 10.1016/j.resp.2015.10.009 26506253 PMC4842164

[B56] Golder F. J. Fuller D. D. Davenport P. W. Johnson R. D. Reier P. J. Bolser D. C. (2003). Respiratory motor recovery after unilateral spinal cord injury: eliminating crossed phrenic activity decreases tidal volume and increases contralateral respiratory motor output. *J. Neurosci.* 23 2494–2501. 10.1523/JNEUROSCI.23-06-02494.2003 12657710 PMC6742041

[B57] Golder F. J. Fuller D. D. Lovett-Barr M. R. Vinit S. Resnick D. K. Mitchell G. S. (2011). Breathing patterns after mid-cervical spinal contusion in rats. *Exp. Neurol.* 231 97–103. 10.1016/j.expneurol.2011.05.020 21683697 PMC3172815

[B58] Golder F. J. Mitchell G. S. (2005). Spinal synaptic enhancement with acute intermittent hypoxia improves respiratory function after chronic cervical spinal cord injury. *J. Neurosci.* 25 2925–2932. 10.1523/JNEUROSCI.0148-05.2005 15772352 PMC6725150

[B59] Golder F. J. Reier P. J. Bolser D. C. (2001). Altered respiratory motor drive after spinal cord injury: supraspinal and bilateral effects of a unilateral lesion. *J. Neurosci.* 21 8680–8689. 10.1523/JNEUROSCI.21-21-08680.2001 11606656 PMC6762779

[B60] Gonzalez-Rothi E. J. Lee K. Z. Dale E. A. Reier P. J. Mitchell G. S. Fuller D. D. (2015). Intermittent hypoxia and neurorehabilitation. *J. Appl. Physiol.* 119 1455–1465. 10.1152/japplphysiol.00235.2015 25997947 PMC4683349

[B61] Gonzalez-Rothi E. J. Tadjalli A. Allen L. L. Ciesla M. C. El Chami M. Mitchell G. (2021). Protocol-specific effects of intermittent hypoxia preconditioning on phrenic motor plasticity in rats with chronic cervical spinal cord injury. *J. Neurotrauma* 38 1292–1305. 10.1089/neu.2020.7324 33446048 PMC8182475

[B62] Goshgarian H. G. (2003). The crossed phrenic phenomenon: a model for plasticity in the respiratory pathways following spinal cord injury. *J. Appl. Physiol.* 94 795–810. 10.1152/japplphysiol.00847.2002 12531916

[B63] Goshgarian H. G. (2009). The crossed phrenic phenomenon and recovery of function following spinal cord injury. *Respir. Physiol. Neurobiol.* 169 85–93. 10.1016/j.resp.2009.06.005 19539790 PMC2783917

[B64] Gransee H. M. Zhan W. Z. Sieck G. C. Mantilla C. B. (2015). Localized delivery of brain-derived neurotrophic factor-expressing mesenchymal stem cells enhances functional recovery following cervical spinal cord injury. *J. Neurotrauma* 32 185–193. 10.1089/neu.2014.3464 25093762 PMC4298751

[B65] Griffin H. S. Pugh K. Kumar P. Balanos G. M. (2012). Long-term facilitation of ventilation following acute continuous hypoxia in awake humans during sustained hypercapnia. *J. Physiol.* 590 5151–5165. 10.1113/jphysiol.2012.236109 22826133 PMC3497569

[B66] Gutierrez D. V. Clark M. Nwanna O. Alilain W. J. (2013). Intermittent hypoxia training after C2 hemisection modifies the expression of PTEN and mTOR. *Exp. Neurol.* 248 45–52. 10.1016/j.expneurol.2013.05.013 23726960

[B67] Guyenet P. G. Stornetta R. L. Abbott S. B. Depuy S. D. Kanbar R. (2012). The retrotrapezoid nucleus and breathing. *Adv. Exp. Med. Biol.* 758 115–122. 10.1007/978-94-007-4584-1_16 23080151 PMC5111164

[B68] Guyenet P. G. Stornetta R. L. Souza G. Abbott S. B. G. Shi Y. Bayliss D. A. (2019). The Retrotrapezoid Nucleus: central Chemoreceptor and Regulator of Breathing Automaticity. *Trends Neurosci.* 42 807–824. 10.1016/j.tins.2019.09.002 31635852 PMC6825900

[B69] Haas F. Axen K. Pineda H. Gandino D. Haas A. (1985). Temporal pulmonary function changes in cervical cord injury. *Arch. Phys. Med. Rehabil.* 66 139–144. 3977564

[B70] Hajela N. Mummidisetty C. K. Smith A. C. Knikou M. (2013). Corticospinal reorganization after locomotor training in a person with motor incomplete paraplegia. *Biomed. Res. Int.* 2013:516427. 10.1155/2013/516427 23484130 PMC3591158

[B71] Harkema S. J. (2001). Neural plasticity after human spinal cord injury: application of locomotor training to the rehabilitation of walking. *Neuroscientist* 7 455–468. 10.1177/107385840100700514 11597104

[B72] Harkema S. J. (2008). Plasticity of interneuronal networks of the functionally isolated human spinal cord. *Brain Res. Rev.* 57 255–264. 10.1016/j.brainresrev.2007.07.012 18042493 PMC2729454

[B73] Harris D. P. Balasubramaniam A. Badr M. S. Mateika J. H. (2006). Long-term facilitation of ventilation and genioglossus muscle activity is evident in the presence of elevated levels of carbon dioxide in awake humans. *Am. J. Physiol. Regul. Integr. Comp. Physiol.* 291 R1111–R1119. 10.1152/ajpregu.00896.2005 16627688

[B74] Hassan A. Arnold B. M. Caine S. Toosi B. M. Verge V. M. K. Muir G. D. (2018). Acute intermittent hypoxia and rehabilitative training following cervical spinal injury alters neuronal hypoxia- and plasticity-associated protein expression. *PLoS One* 13:e0197486. 10.1371/journal.pone.0197486 29775479 PMC5959066

[B75] Hayashi F. Coles S. K. Bach K. B. Mitchell G. S. Mccrimmon D. R. (1993). Time-dependent phrenic nerve responses to carotid afferent activation: intact vs. decerebellate rats. *Am. J. Physiol.* 265 R811–R819. 10.1152/ajpregu.1993.265.4.R811 8238451

[B76] Hayes H. B. Jayaraman A. Herrmann M. Mitchell G. S. Rymer W. Z. Trumbower R. D. (2014). Daily intermittent hypoxia enhances walking after chronic spinal cord injury: a randomized trial. *Neurology* 82 104–113. 10.1212/01.WNL.0000437416.34298.43 24285617 PMC3897437

[B77] Hernandez-Torres V. Gransee H. M. Mantilla C. B. Wang Y. Zhan W. Z. Sieck G. C. (2016). BDNF Effects on Functional Recovery across Motor Behaviors after Cervical Spinal Cord Injury. *J. Neurophysiol*. 117 537–544. 27832605 10.1152/jn.00654.2016 PMC5288474

[B78] Hernandez-Torres V. Gransee H. M. Mantilla C. B. Wang Y. Zhan W. Z. Sieck G. C. (2017). BDNF effects on functional recovery across motor behaviors after cervical spinal cord injury. *J. Neurophysiol.* 117 537–544. 10.1152/jn.00654.2016 27832605 PMC5288474

[B79] Hicks A. L. Ginis K. A. (2008). Treadmill training after spinal cord injury: it’s not just about the walking. *J. Rehabil. Res. Dev.* 45 241–248. 10.1682/JRRD.2007.02.0022 18566942

[B80] Hillen B. K. Abbas J. J. Jung R. (2013). Accelerating locomotor recovery after incomplete spinal injury. *Ann. N. Y. Acad. Sci.* 1279 164–174. 10.1111/nyas.12061 23531014 PMC3616515

[B81] Hodges M. R. Richerson G. B. (2010). The role of medullary serotonin (5-HT) neurons in respiratory control: contributions to eupneic ventilation, CO2 chemoreception, and thermoregulation. *J. Appl. Physiol.* 108 1425–1432. 10.1152/japplphysiol.01270.2009 20133432 PMC2867541

[B82] Hoh D. J. Mercier L. M. Hussey S. P. Lane M. A. (2013). Respiration following spinal cord injury: evidence for human neuroplasticity. *Respir. Physiol. Neurobiol.* 189 450–464. 10.1016/j.resp.2013.07.002 23891679 PMC3815640

[B83] Hopman M. T. Van Der Woude L. H. Dallmeijer A. J. Snoek G. Folgering H. T. (1997). Respiratory muscle strength and endurance in individuals with tetraplegia. *Spinal Cord* 35 104–108. 10.1038/sj.sc.3100353 9044518

[B84] Hormigo K. M. Zholudeva L. V. Spruance V. M. Marchenko V. Cote M. P. Vinit S. (2017). Enhancing neural activity to drive respiratory plasticity following cervical spinal cord injury. *Exp. Neurol.* 287 276–287. 10.1016/j.expneurol.2016.08.018 27582085 PMC5121051

[B85] Houle J. D. Cote M. P. (2013). Axon regeneration and exercise-dependent plasticity after spinal cord injury. *Ann. N. Y. Acad. Sci.* 1279 154–163. 10.1111/nyas.12052 23531013 PMC3616327

[B86] Hsu S. H. Lee K. Z. (2015). Effects of serotonergic agents on respiratory recovery after cervical spinal injury. *J. Appl. Physiol.* 119 1075–1087. 10.1152/japplphysiol.00329.2015 26359482

[B87] Hubli M. Dietz V. (2013). The physiological basis of neurorehabilitation–locomotor training after spinal cord injury. *J. Neuroeng. Rehabil.* 10:5. 10.1186/1743-0003-10-5 23336934 PMC3584845

[B88] Imamura T. (1967). [The effect of auxiliary respiratory muscular training on breathing exercise in cervical cord injuries]. *Kumamoto Igakkai Zasshi* 41 130–151. 5630470

[B89] Iscoe S. Polosa C. (1976). Synchronization of respiratory frequency by somatic afferent stimulation. *J. Appl. Physiol.* 40 138–148. 10.1152/jappl.1976.40.2.138 1248992

[B90] Jack L. P. Purcell M. Allan D. B. Hunt K. J. (2011). The metabolic cost of passive walking during robotics-assisted treadmill exercise. *Technol. Health Care* 19 21–27. 10.3233/THC-2011-0608 21248409

[B91] Jackson A. B. Groomes T. E. (1994). Incidence of respiratory complications following spinal cord injury. *Arch. Phys. Med. Rehabil.* 75 270–275. 10.1016/0003-9993(94)90027-2 8129577

[B92] Jaiswal P. B. Tester N. J. Davenport P. W. (2016). Effect of acute intermittent hypoxia treatment on ventilatory load compensation and magnitude estimation of inspiratory resistive loads in an individual with chronic incomplete cervical spinal cord injury. *J. Spinal Cord Med.* 39 103–110. 10.1179/2045772314Y.0000000277 25400130 PMC4725779

[B93] Jensen V. N. Alilain W. J. Crone S. A. (2019). Role of Propriospinal Neurons in Control of Respiratory Muscles and Recovery of Breathing Following Injury. *Front. Syst. Neurosci.* 13:84. 10.3389/fnsys.2019.00084 32009911 PMC6978673

[B94] Jouett N. P. Watenpaugh D. E. Dunlap M. E. Smith M. L. (2015). Interactive effects of hypoxia, hypercapnia and lung volume on sympathetic nerve activity in humans. *Exp. Physiol.* 100 1018–1029. 10.1113/EP085092 26132990

[B95] Kaur S. De Luca R. Khanday M. A. Bandaru S. S. Thomas R. C. Broadhurst R. Y. (2020). Role of serotonergic dorsal raphe neurons in hypercapnia-induced arousals. *Nat. Commun.* 11:2769. 10.1038/s41467-020-16518-9 32488015 PMC7265411

[B96] Kim C. Y. Lee J. S. Kim H. D. Lee D. J. (2017). Short-term effects of respiratory muscle training combined with the abdominal drawing-in maneuver on the decreased pulmonary function of individuals with chronic spinal cord injury: a pilot randomized controlled trial. *J. Spinal Cord Med.* 40 17–25. 10.1080/10790268.2016.1198576 27463071 PMC5376135

[B97] Kinkead R. Bach K. B. Johnson S. M. Hodgeman B. A. Mitchell G. S. (2001). Plasticity in respiratory motor control: intermittent hypoxia and hypercapnia activate opposing serotonergic and noradrenergic modulatory systems. *Comp. Biochem. Physiol. A Mol. Integr. Physiol.* 130 207–218. 10.1016/S1095-6433(01)00393-2 11544068

[B98] Komnenov D. Solarewicz J. Z. Afzal F. Nantwi K. D. Kuhn D. M. Mateika J. H. (2016). Intermittent hypoxia promotes recovery of respiratory motor function in spinal cord-injured mice depleted of serotonin in the central nervous system. *J. Appl. Physiol.* 121 545–557. 10.1152/japplphysiol.00448.2016 27402561

[B99] Laciuga H. Rosenbek J. C. Davenport P. W. Sapienza C. M. (2014). Functional outcomes associated with expiratory muscle strength training: narrative review. *J. Rehabil. Res. Dev.* 51 535–546. 10.1682/JRRD.2013.03.0076 25144167

[B100] Lane M. A. (2011). Spinal respiratory motoneurons and interneurons. *Respir. Physiol. Neurobiol.* 179 3–13. 10.1016/j.resp.2011.07.004 21782981

[B101] Lane M. A. Fuller D. D. White T. E. Reier P. J. (2008a). Respiratory neuroplasticity and cervical spinal cord injury: translational perspectives. *Trends Neurosci.* 31 538–547. 10.1016/j.tins.2008.07.002 18775573 PMC2577878

[B102] Lane M. A. White T. E. Coutts M. A. Jones A. L. Sandhu M. S. Bloom D. C. (2008b). Cervical prephrenic interneurons in the normal and lesioned spinal cord of the adult rat. *J. Comp. Neurol.* 511 692–709. 10.1002/cne.21864 18924146 PMC2597676

[B103] Lane M. A. Lee K. Z. Fuller D. D. Reier P. J. (2009). Spinal circuitry and respiratory recovery following spinal cord injury. *Respir. Physiol. Neurobiol.* 169 123–132. 10.1016/j.resp.2009.08.007 19698805 PMC2783531

[B104] Lane M. A. Lee K. Z. Salazar K. O’steen B. E. Bloom D. C. Fuller D. D. (2012). Respiratory function following bilateral mid-cervical contusion injury in the adult rat. *Exp. Neurol.* 235 197–210. 10.1016/j.expneurol.2011.09.024 21963673 PMC3270207

[B105] Ledlie J. F. Kelsen S. G. Cherniack N. S. Fishman A. P. (1981). Effects of hypercapnia and hypoxia on phrenic nerve activity and respiratory timing. *J. Appl. Physiol. Respir. Environ. Exerc. Physiol.* 51 732–738. 10.1152/jappl.1981.51.3.732 7327975

[B106] Ledsome J. R. Sharp J. M. (1981). Pulmonary function in acute cervical cord injury. *Am. Rev. Respir. Dis.* 124 41–44. 7258818 10.1164/arrd.1981.124.1.41

[B107] Lee K. Z. Chiang S. C. Li Y. J. (2017). Mild Acute Intermittent Hypoxia Improves Respiratory Function in Unanesthetized Rats With Midcervical Contusion. *Neurorehabil. Neural Repair* 31 364–375. 10.1177/1545968316680494 28332435

[B108] Legg Ditterline B. E. Aslan S. C. Randall D. C. Harkema S. J. Castillo C. Ovechkin A. V. (2018). Effects of Respiratory Training on Heart Rate Variability and Baroreflex Sensitivity in Individuals With Chronic Spinal Cord Injury. *Arch. Phys. Med. Rehabil.* 99 423–432. 10.1016/j.apmr.2017.06.033 28802811 PMC5807237

[B109] Levine S. Nguyen T. Taylor N. Friscia M. E. Budak M. T. Rothenberg P. (2008). Rapid disuse atrophy of diaphragm fibers in mechanically ventilated humans. *N. Engl. J. Med.* 358 1327–1335. 10.1056/NEJMoa070447 18367735

[B110] Lewis L. J. Brookhart J. M. (1951). Significance of the crossed phrenic phenomenon. *Am. J. Physiol.* 166 241–254. 10.1152/ajplegacy.1951.166.2.241 14857171

[B111] Lin M. T. Vinit S. Lee K. Z. (2021). Functional role of carbon dioxide on intermittent hypoxia induced respiratory response following mid-cervical contusion in the rat. *Exp. Neurol.* 339:113610. 10.1016/j.expneurol.2021.113610 33453216

[B112] Ling L. Fuller D. D. Bach K. B. Kinkead R. Olson E. B. Jr. Mitchell G. S. (2001). Chronic intermittent hypoxia elicits serotonin-dependent plasticity in the central neural control of breathing. *J. Neurosci.* 21 5381–5388. 10.1523/JNEUROSCI.21-14-05381.2001 11438615 PMC6762841

[B113] Linn W. S. Adkins R. H. Gong H. Jr. Waters R. L. (2000). Pulmonary function in chronic spinal cord injury: a cross-sectional survey of 222 southern California adult outpatients. *Arch. Phys. Med. Rehabil.* 81 757–763. 10.1016/S0003-9993(00)90107-2 10857520

[B114] Long W. Lobchuk D. Anthonisen N. R. (1994). Ventilatory responses to CO2 and hypoxia after sustained hypoxia in awake cats. *J. Appl. Physiol.* 76 2262–2266. 10.1152/jappl.1994.76.6.2262 7928845

[B115] Lovett-Barr M. R. Satriotomo I. Muir G. D. Wilkerson J. E. Hoffman M. S. Vinit S. (2012). Repetitive intermittent hypoxia induces respiratory and somatic motor recovery after chronic cervical spinal injury. *J. Neurosci.* 32 3591–3600. 10.1523/JNEUROSCI.2908-11.2012 22423083 PMC3349282

[B116] Lu P. Jones L. L. Tuszynski M. H. (2005). BDNF-expressing marrow stromal cells support extensive axonal growth at sites of spinal cord injury. *Exp. Neurol.* 191 344–360. 10.1016/j.expneurol.2004.09.018 15649491

[B117] Lynch M. Duffell L. Sandhu M. Srivatsan S. Deatsch K. Kessler A. (2017). Effect of acute intermittent hypoxia on motor function in individuals with chronic spinal cord injury following ibuprofen pretreatment: a pilot study. *J. Spinal Cord Med.* 40 295–303. 10.1080/10790268.2016.1142137 26856344 PMC5472017

[B118] Lynskey J. V. Belanger A. Jung R. (2008). Activity-dependent plasticity in spinal cord injury. *J. Rehabil. Res. Dev.* 45 229–240. 10.1682/JRRD.2007.03.0047 18566941 PMC2562625

[B119] Mantilla C. B. Gransee H. M. Zhan W. Z. Sieck G. C. (2013). Motoneuron BDNF/TrkB signaling enhances functional recovery after cervical spinal cord injury. *Exp. Neurol.* 247 101–109. 10.1016/j.expneurol.2013.04.002 23583688 PMC3742616

[B120] Mantilla C. B. Greising S. M. Stowe J. M. Zhan W. Z. Sieck G. C. (2014). TrkB kinase activity is critical for recovery of respiratory function after cervical spinal cord hemisection. *Exp. Neurol.* 261 190–195. 10.1016/j.expneurol.2014.05.027 24910201 PMC4194245

[B121] Mantilla C. B. Sieck G. C. (2009). Neuromuscular adaptations to respiratory muscle inactivity. *Respir. Physiol. Neurobiol.* 169 133–140. 10.1016/j.resp.2009.09.002 19744580 PMC2783688

[B122] Martin A. D. Smith B. K. Davenport P. D. Harman E. Gonzalez-Rothi R. J. Baz M. (2011). Inspiratory muscle strength training improves weaning outcome in failure to wean patients: a randomized trial. *Crit. Care* 15:R84. 10.1186/cc10081 21385346 PMC3219341

[B123] Martinez M. Delivet-Mongrain H. Rossignol S. (2013). Treadmill training promotes spinal changes leading to locomotor recovery after partial spinal cord injury in cats. *J. Neurophysiol.* 109 2909–2922. 10.1152/jn.01044.2012 23554433

[B124] Martinez-Galvez G. Zambrano J. M. Diaz Soto J. C. Zhan W. Z. Gransee H. M. Sieck G. C. (2016). TrkB gene therapy by adeno-associated virus enhances recovery after cervical spinal cord injury. *Exp. Neurol.* 276 31–40. 10.1016/j.expneurol.2015.11.007 26607912 PMC4715974

[B125] Millhorn D. E. Eldridge F. L. (1986). Role of ventrolateral medulla in regulation of respiratory and cardiovascular systems. *J. Appl. Physiol.* 61 1249–1263. 10.1152/jappl.1986.61.4.1249 3536832

[B126] Millhorn D. E. Eldridge F. L. Waldrop T. G. (1980). Prolonged stimulation of respiration by a new central neural mechanism. *Respir. Physiol.* 41 87–103. 10.1016/0034-5687(80)90025-0 6771859

[B127] Mitchell G. S. Baker T. L. Nanda S. A. Fuller D. D. Zabka A. G. Hodgeman B. A. (2001). Invited review: intermittent hypoxia and respiratory plasticity. *J. Appl. Physiol.* 90 2466–2475. 10.1152/jappl.2001.90.6.2466 11356815

[B128] Mitchell G. S. Johnson S. M. (2003). Neuroplasticity in respiratory motor control. *J. Appl. Physiol.* 94 358–374. 10.1152/japplphysiol.00523.2002 12486024

[B129] Molkov Y. I. Shevtsova N. A. Park C. Ben-Tal A. Smith J. C. Rubin J. E. (2014). A closed-loop model of the respiratory system: focus on hypercapnia and active expiration. *PLoS One* 9:e109894. 10.1371/journal.pone.0109894 25302708 PMC4193835

[B130] Morawietz C. Moffat F. (2013). Effects of locomotor training after incomplete spinal cord injury: a systematic review. *Arch. Phys. Med. Rehabil.* 94 2297–2308. 10.1016/j.apmr.2013.06.023 23850614

[B131] Morin D. Viala D. (2002). Coordinations of locomotor and respiratory rhythms in vitro are critically dependent on hindlimb sensory inputs. *J. Neurosci.* 22 4756–4765. 10.1523/JNEUROSCI.22-11-04756.2002 12040083 PMC6758812

[B132] Morris K. F. Arata A. Shannon R. Lindsey B. G. (1996). Long-term facilitation of phrenic nerve activity in cats: responses and short time scale correlations of medullary neurones. *J. Physiol.* 490 463–480. 10.1113/jphysiol.1996.sp021158 8821143 PMC1158683

[B133] Mueller G. Perret C. Spengler C. M. (2006). Optimal intensity for respiratory muscle endurance training in patients with spinal cord injury. *J. Rehabil. Med.* 38 381–386. 10.1080/16501970600780369 17067972

[B134] Nair J. Bezdudnaya T. Zholudeva L. V. Detloff M. R. Reier P. J. Lane M. A. (2017). Histological identification of phrenic afferent projections to the spinal cord. *Respir. Physiol. Neurobiol.* 236 57–68. 10.1016/j.resp.2016.11.006 27838334 PMC6126536

[B135] Nattie E. Li A. (2012). Central chemoreceptors: locations and functions. *Compr. Physiol.* 2 221–254. 10.1002/cphy.c100083 23728974 PMC4802370

[B136] Navarrete-Opazo A. Alcayaga J. Sepulveda O. Rojas E. Astudillo C. (2017a). Repetitive Intermittent Hypoxia and Locomotor Training Enhances Walking Function in Incomplete Spinal Cord Injury Subjects: a Randomized, Triple-Blind, Placebo-Controlled Clinical Trial. *J. Neurotrauma* 34 1803–1812. 10.1089/neu.2016.4478 27329506

[B137] Navarrete-Opazo A. Alcayaga J. J. Sepulveda O. Varas G. (2017b). Intermittent Hypoxia and Locomotor Training Enhances Dynamic but Not Standing Balance in Patients With Incomplete Spinal Cord Injury. *Arch. Phys. Med. Rehabil.* 98 415–424. 10.1016/j.apmr.2016.09.114 27702556

[B138] Navarrete-Opazo A. Dougherty B. J. Mitchell G. S. (2017c). Enhanced recovery of breathing capacity from combined adenosine 2A receptor inhibition and daily acute intermittent hypoxia after chronic cervical spinal injury. *Exp. Neurol.* 287 93–101. 10.1016/j.expneurol.2016.03.026 27079999 PMC5193117

[B139] Navarrete-Opazo A. Alcayaga J. Testa D. Quinteros A. L. (2016). Intermittent Hypoxia Does not Elicit Memory Impairment in Spinal Cord Injury Patients. *Arch. Clin. Neuropsychol.* 31 332–342. 10.1093/arclin/acw012 27084733

[B140] Navarrete-Opazo A. Mitchell G. S. (2014). Therapeutic potential of intermittent hypoxia: a matter of dose. *Am. J. Physiol. Regul. Integr. Comp. Physiol.* 307 R1181–R1197. 10.1152/ajpregu.00208.2014 25231353 PMC4315448

[B141] Navarrete-Opazo A. Vinit S. Dougherty B. J. Mitchell G. S. (2015). Daily acute intermittent hypoxia elicits functional recovery of diaphragm and inspiratory intercostal muscle activity after acute cervic al spinal injury. *Exp. Neurol.* 266 1–10. 10.1016/j.expneurol.2015.02.007 25687551 PMC4716671

[B142] Navarrete-Opazo A. A. Vinit S. Mitchell G. S. (2014). Adenosine 2A receptor inhibition enhances intermittent hypoxia-induced diaphragm but not intercostal long-term facilitation. *J. Neurotrauma* 31 1975–1984. 10.1089/neu.2014.3393 25003645 PMC4245839

[B143] Nicaise C. Frank D. M. Hala T. J. Authelet M. Pochet R. Adriaens D. (2013). Early phrenic motor neuron loss and transient respiratory abnormalities after unilateral cervical spinal cord contusion. *J. Neurotrauma* 30 1092–1099. 10.1089/neu.2012.2728 23534670 PMC3689927

[B144] Nicaise C. Hala T. J. Frank D. M. Parker J. L. Authelet M. Leroy K. (2012a). Phrenic motor neuron degeneration compromises phrenic axonal circuitry and diaphragm activity in a unilateral cervical contusion model of spinal cord injury. *Exp. Neurol.* 235 539–552. 10.1016/j.expneurol.2012.03.007 22465264

[B145] Nicaise C. Putatunda R. Hala T. J. Regan K. A. Frank D. M. Brion J. P. (2012b). Degeneration of phrenic motor neurons induces long-term diaphragm deficits following mid-cervical spinal contusion in mice. *J. Neurotrauma* 29 2748–2760. 10.1089/neu.2012.2467 23176637 PMC3521144

[B146] Nielsen A. M. Bisgard G. E. Mitchell G. S. (1986). Phrenic nerve responses to hypoxia and CO2 in decerebrate dogs. *Respir. Physiol.* 65 267–283. 10.1016/0034-5687(86)90012-5 3097770

[B147] O’Hara T. E. Jr. Goshgarian H. G. (1991). Quantitative assessment of phrenic nerve functional recovery mediated by the crossed phrenic reflex at various time intervals after spinal cord injury. *Exp. Neurol.* 111 244–250. 10.1016/0014-4886(91)90012-2 1989900

[B148] Onders R. Mcgee M. F. Marks J. Chak A. Schilz R. Rosen M. J. (2007). Diaphragm pacing with natural orifice transluminal endoscopic surgery: potential for difficult-to-wean intensive care unit patients. *Surg. Endosc.* 21 475–479. 10.1007/s00464-006-9125-4 17177078

[B149] Oza C. S. Giszter S. F. (2014). Plasticity and alterations of trunk motor cortex following spinal cord injury and non-stepping robot and treadmill training. *Exp. Neurol.* 256 57–69. 10.1016/j.expneurol.2014.03.012 24704619 PMC7222855

[B150] Oza C. S. Giszter S. F. (2015). Trunk robot rehabilitation training with active stepping reorganizes and enriches trunk motor cortex representations in spinal transected rats. *J. Neurosci.* 35 7174–7189. 10.1523/JNEUROSCI.4366-14.2015 25948267 PMC4420783

[B151] Persegol L. Palisses R. Viala D. (1993). Characterization of hindlimb muscle afferents involved in ventilatory effects observed in decerebrate and spinal preparations. *Exp. Brain Res.* 92 495–501. 10.1007/BF00229038 8454012

[B152] Porter W. T. (1895). The Path of the Respiratory Impulse from the Bulb to the Phrenic Nuclei. *J. Physiol.* 17 455–485. 10.1113/jphysiol.1895.sp000553 16992199 PMC1514646

[B153] Postma K. Vlemmix L. Y. Haisma J. A. De Groot S. Sluis T. A. Stam H. J. (2015). Longitudinal association between respiratory muscle strength and cough capacity in persons with spinal cord injury: an explorative analysis of data from a randomized controlled trial. *J. Rehabil. Med.* 47 722–726. 10.2340/16501977-1986 26074331

[B154] Potts J. T. Rybak I. A. Paton J. F. (2005). Respiratory rhythm entrainment by somatic afferent stimulation. *J. Neurosci.* 25 1965–1978. 10.1523/JNEUROSCI.3881-04.2005 15728836 PMC6726065

[B155] Powers S. K. Shanely R. A. Coombes J. S. Koesterer T. J. Mckenzie M. Van Gammeren D. (2002). Mechanical ventilation results in progressive contractile dysfunction in the diaphragm. *J. Appl. Physiol.* 92 1851–1858. 10.1152/ajpregu.00231.2013 11960933

[B156] Powers S. K. Wiggs M. P. Sollanek K. J. Smuder A. J. (2013). Ventilator-induced diaphragm dysfunction: cause and effect. *Am. J. Physiol. Regul. Integr. Comp. Physiol.* 305 R464–R477. 23842681 10.1152/ajpregu.00231.2013

[B157] Prabhakar N. R. Peng Y. J. Jacono F. J. Kumar G. K. Dick T. E. (2005). Cardiovascular alterations by chronic intermittent hypoxia: importance of carotid body chemoreflexes. *Clin. Exp. Pharmacol. Physiol.* 32 447–449. 10.1111/j.1440-1681.2005.04209.x 15854156

[B158] Prosser-Loose E. J. Hassan A. Mitchell G. S. Muir G. D. (2015). Delayed Intervention with Intermittent Hypoxia and Task Training Improves Forelimb Function in a Rat Model of Cervical Spinal Injury. *J. Neurotrauma* 32 1403–1412. 10.1089/neu.2014.3789 25664481

[B159] Raab A. M. Krebs J. Pfister M. Perret C. Hopman M. Mueller G. (2019). Respiratory muscle training in individuals with spinal cord injury: effect of training intensity and -volume on improvements in respiratory muscle strength. *Spinal Cord* 57 482–489. 10.1038/s41393-019-0249-5 30700854

[B160] Raineteau O. Schwab M. E. (2001). Plasticity of motor systems after incomplete spinal cord injury. *Nat. Rev. Neurosci.* 2 263–273. 10.1038/35067570 11283749

[B161] Rank M. M. Flynn J. R. Battistuzzo C. R. Galea M. P. Callister R. Callister R. J. (2015). Functional changes in deep dorsal horn interneurons following spinal cord injury are enhanced with different durations of exercise training. *J. Physiol.* 593 331–345. 10.1113/jphysiol.2014.282640 25556804 PMC4293071

[B162] Rhea M. R. Alvar B. A. Burkett L. N. (2002). Single versus multiple sets for strength: a meta-analysis to address the controversy. *Res. Q. Exerc. Sport* 73 485–488. 10.1080/02701367.2002.10609050 12495252

[B163] Roth E. J. Stenson K. W. Powley S. Oken J. Primack S. Nussbaum S. B. (2010). Expiratory muscle training in spinal cord injury: a randomized controlled trial. *Arch. Phys. Med. Rehabil.* 91 857–861. 10.1016/j.apmr.2010.02.012 20510974

[B164] Row B. W. (2007). Intermittent hypoxia and cognitive function: implications from chronic animal models. *Adv. Exp. Med. Biol.* 618 51–67. 10.1007/978-0-387-75434-5_5 18269188

[B165] Sandhu M. S. Baekey D. M. Maling N. G. Sanchez J. C. Reier P. J. Fuller D. D. (2015). Midcervical neuronal discharge patterns during and following hypoxia. *J. Neurophysiol.* 113 2091–2101. 10.1152/jn.00834.2014 25552641 PMC4416573

[B166] Sandhu M. S. Dougherty B. J. Lane M. A. Bolser D. C. Kirkwood P. A. Reier P. J. (2009). Respiratory recovery following high cervical hemisection. *Respir. Physiol. Neurobiol.* 169 94–101. 10.1016/j.resp.2009.06.014 19560562 PMC2783827

[B167] Sandhu M. S. Gray E. Kocherginsky M. Jayaraman A. Mitchell G. S. Rymer W. Z. (2019). Prednisolone Pretreatment Enhances Intermittent Hypoxia-Induced Plasticity in Persons With Chronic Incomplete Spinal Cord Injury. *Neurorehabil. Neural Repair* 33 911–921. 10.1177/1545968319872992 31524075

[B168] Sandhu M. S. Perez M. A. Oudega M. Mitchell G. S. Rymer W. Z. (2021). Efficacy and time course of acute intermittent hypoxia effects in the upper extremities of people with cervical spinal cord injury. *Exp. Neurol.* 342:113722. 10.1016/j.expneurol.2021.113722 33932397 PMC8530358

[B169] Sankari A. Bascom A. T. Riehani A. Badr M. S. (2015). Tetraplegia is associated with enhanced peripheral chemoreflex sensitivity and ventilatory long-term facilitation. *J. Appl. Physiol.* 119 1183–1193. 10.1152/japplphysiol.00088.2015 26272316 PMC4758675

[B170] Sapienza C. Troche M. Pitts T. Davenport P. (2011). Respiratory strength training: concept and intervention outcomes. *Semin. Speech Lang.* 32 21–30. 10.1055/s-0031-1271972 21491356

[B171] Sapienza C. M. Wheeler K. (2006). Respiratory muscle strength training: functional outcomes versus plasticity. *Semin. Speech Lang.* 27 236–244. 10.1055/s-2006-955114 17117350

[B172] Satriotomo I. Nichols N. L. Dale E. A. Emery A. T. Dahlberg J. M. Mitchell G. S. (2016). Repetitive acute intermittent hypoxia increases growth/neurotrophic factor expression in non-respiratory motor neurons. *Neuroscience* 322 479–488. 10.1016/j.neuroscience.2016.02.060 26944605 PMC5203934

[B173] Schlumberger A. Stec J. Schmidtbleicher D. (2001). Single- vs. multiple-set strength training in women. *J. Strength Cond. Res.* 15 284–289. 10.1519/00124278-200108000-00004 11710652

[B174] Serebrovskaya T. V. Xi L. (2016). Intermittent hypoxia training as non-pharmacologic therapy for cardiovascular diseases: practical analysis on methods and equipment. *Exp. Biol. Med.* 241 1708–1723. 10.1177/1535370216657614 27407098 PMC4999622

[B175] Sherman M. F. Lam T. Sheel A. W. (2009). Locomotor-respiratory synchronization after body weight supported treadmill training in incomplete tetraplegia: a case report. *Spinal Cord* 47 896–898. 10.1038/sc.2009.50 19451913

[B176] Shigemura M. Welch L. C. Sznajder J. I. (2020). Hypercapnia Regulates Gene Expression and Tissue Function. *Front. Physiol.* 11:598122. 10.3389/fphys.2020.598122 33329047 PMC7715027

[B177] Shin J. C. Han E. Y. Cho K. H. Im S. H. (2019). Improvement in Pulmonary Function with Short-term Rehabilitation Treatment in Spinal Cord Injury Patients. *Sci. Rep.* 9:17091. 10.1038/s41598-019-52526-6 31745108 PMC6863911

[B178] Sieck G. C. Mantilla C. B. (2009). Role of neurotrophins in recovery of phrenic motor function following spinal cord injury. *Respir. Physiol. Neurobiol.* 169 218–225. 10.1016/j.resp.2009.08.008 19703592 PMC2783678

[B179] Singh A. Balasubramanian S. Murray M. Lemay M. Houle J. (2011a). Role of spared pathways in locomotor recovery after body-weight-supported treadmill training in contused rats. *J. Neurotrauma* 28 2405–2416. 10.1089/neu.2010.1660 21568686 PMC3235344

[B180] Singh A. Murray M. Houle J. D. (2011b). A training paradigm to enhance motor recovery in contused rats: effects of staircase training. *Neurorehabil. Neural Repair* 25 24–34. 10.1177/1545968310378510 20858910

[B181] Smith B. K. Gabrielli A. Davenport P. W. Martin A. D. (2014). Effect of training on inspiratory load compensation in weaned and unweaned mechanically ventilated ICU patients. *Respir. Care* 59 22–31. 10.4187/respcare.02053 23764858 PMC4157996

[B182] Smith H. R. Leibold N. K. Rappoport D. A. Ginapp C. M. Purnell B. S. Bode N. M. (2018). Dorsal Raphe Serotonin Neurons Mediate CO2-Induced Arousal from Sleep. *J. Neurosci.* 38 1915–1925. 10.1523/JNEUROSCI.2182-17.2018 29378860 PMC5824737

[B183] Smuder A. J. Gonzalez-Rothi E. J. Kwon O. S. Morton A. B. Sollanek K. J. Powers S. K. (2016). Cervical spinal cord injury exacerbates ventilator-induced diaphragm dysfunction. *J. Appl. Physiol.* 120 166–177. 10.1152/japplphysiol.00488.2015 26472866 PMC4719055

[B184] Somers V. K. Mark A. L. Zavala D. C. Abboud F. M. (1989). Contrasting effects of hypoxia and hypercapnia on ventilation and sympathetic activity in humans. *J. Appl. Physiol.* 67 2101–2106. 10.1152/jappl.1989.67.5.2101 2513316

[B185] Stipica I. Pavlinac Dodig I. Pecotic R. Dogas Z. Valic Z. Valic M. (2016). Periodicity during hypercapnic and hypoxic stimulus is crucial in distinct aspects of phrenic nerve plasticity. *Physiol. Res.* 65 133–143. 10.33549/physiolres.933012 26596313

[B186] Stipica Safic I. Pecotic R. Pavlinac Dodig I. Dogas Z. Valic Z. Valic M. (2018). Phrenic long-term depression evoked by intermittent hypercapnia is modulated by serotonergic and adrenergic receptors in raphe nuclei. *J. Neurophysiol.* 120 321–329. 10.1152/jn.00776.2017 29617215

[B187] Streeter K. A. Sunshine M. D. Patel S. Gonzalez-Rothi E. J. Reier P. J. Baekey D. M. (2017). Intermittent Hypoxia Enhances Functional Connectivity of Midcervical Spinal Interneurons. *J. Neurosci.* 37 8349–8362. 10.1523/JNEUROSCI.0992-17.2017 28751456 PMC5577852

[B188] Sutor T. Cavka K. Vose A. K. Welch J. F. Davenport P. Fuller D. D. (2021). Single-session effects of acute intermittent hypoxia on breathing function after human spinal cord injury. *Exp. Neurol.* 342:113735. 10.1016/j.expneurol.2021.113735 33951477 PMC8616729

[B189] Tan A. Q. Sohn W. J. Naidu A. Trumbower R. D. (2021). Daily acute intermittent hypoxia combined with walking practice enhances walking performance but not intralimb motor coordination in persons with chronic incomplete spinal cord injury. *Exp. Neurol.* 340:113669. 10.1016/j.expneurol.2021.113669 33647273 PMC8119335

[B190] Tasali E. Ip M. S. (2008). Obstructive sleep apnea and metabolic syndrome: alterations in glucose metabolism and inflammation. *Proc. Am. Thorac. Soc.* 5 207–217. 10.1513/pats.200708-139MG 18250214

[B191] Teppema L. J. Berkenbosch A. Veening J. G. Olievier C. N. (1994). Hypercapnia induces c-fos expression in neurons of retrotrapezoid nucleus in cats. *Brain Res.* 635 353–356. 10.1016/0006-8993(94)91462-1 8173977

[B192] Teppema L. J. Veening J. G. Kranenburg A. Dahan A. Berkenbosch A. Olievier C. (1997). Expression of c-fos in the rat brainstem after exposure to hypoxia and to normoxic and hyperoxic hypercapnia. *J. Comp. Neurol.* 388 169–190. 10.1002/(SICI)1096-9861(19971117)388:2<169::AID-CNE1>3.0.CO;2-# 9368836

[B193] Terson de Paleville D. G. Mckay W. B. Folz R. J. Ovechkin A. V. (2011). Respiratory motor control disrupted by spinal cord injury: mechanisms, evaluation, and restoration. *Transl. Stroke Res.* 2 463–473. 10.1007/s12975-011-0114-0 22408690 PMC3297359

[B194] Terson de Paleville D. Mckay W. Aslan S. Folz R. Sayenko D. Ovechkin A. (2013). Locomotor step training with body weight support improves respiratory motor function in individuals with chronic spinal cord injury. *Respir. Physiol. Neurobiol.* 189 491–497. 10.1016/j.resp.2013.08.018 23999001 PMC3833892

[B195] Tester N. J. Fuller D. D. Fromm J. S. Spiess M. R. Behrman A. L. Mateika J. H. (2014). Long-term facilitation of ventilation in humans with chronic spinal cord injury. *Am. J. Respir. Crit. Care Med.* 189 57–65. 10.1164/rccm.201401-0089LE 24224903 PMC3919124

[B196] Tow A. M. Graves D. E. Carter R. E. (2001). Vital capacity in tetraplegics twenty years and beyond. *Spinal Co rd* 39 139–144. 10.1038/sj.sc.3101136 11326323

[B197] Trumbower R. D. Hayes H. B. Mitchell G. S. Wolf S. L. Stahl V. A. (2017). Effects of acute intermittent hypoxia on hand use after spinal cord trauma: a preliminary study. *Neurology* 89 1904–1907. 10.1212/WNL.0000000000004596 28972191 PMC5664298

[B198] Trumbower R. D. Jayaraman A. Mitchell G. S. Rymer W. Z. (2012). Exposure to acute intermittent hypoxia augments somatic motor function in humans with incomplete spinal cord injury. *Neurorehabil. Neural Repair* 26 163–172. 10.1177/1545968311412055 21821826

[B199] Turner S. M. Elmallah M. K. Hoyt A. K. Greer J. J. Fuller D. D. (2016). Ampakine CX717 potentiates intermittent hypoxia-induced hypoglossal long-term facilitation. *J. Neurophysiol.* 116 1232–1238. 10.1152/jn.00210.2016 27306673 PMC5018053

[B200] Valic M. Pecotic R. Pavlinac Dodig I. Valic Z. Stipica I. Dogas Z. (2016). Intermittent hypercapnia-induced phrenic long-term depression is revealed after serotonin receptor blockade with methysergide in anaesthetized rats. *Exp. Physiol.* 101 319–331. 10.1113/EP085161 26621042

[B201] van den Brand R. Heutschi J. Barraud Q. Digiovanna J. Bartholdi K. Huerlimann M. (2012). Restoring voluntary control of locomotion after paralyzing spinal cord injury. *Science* 336 1182–1185. 10.1126/science.1217416 22654062

[B202] Van Houtte S. Vanlandewijck Y. Gosselink R. (2006). Respiratory muscle training in persons with spinal cord injury: a systematic review. *Respir. Med.* 100 1886–1895. 10.1016/j.rmed.2006.02.029 16626951

[B203] Vermeulen T. D. Benbaruj J. Brown C. V. Shafer B. M. Floras J. S. Foster G. E. (2020). Peripheral chemoreflex contribution to ventilatory long-term facilitation induced by acute intermittent hypercapnic hypoxia in males and females. *J. Physiol.* 598 4713–4730. 10.1113/JP280458 32744340

[B204] Vinit S. Boulenguez P. Efthimiadi L. Stamegna J. C. Gauthier P. Kastner A. (2005). Axotomized bulbospinal neurons express c-Jun after cervical spinal cord injury. *Neuroreport* 16 1535–1539. 10.1097/01.wnr.0000179075.32035.0f 16148740

[B205] Vinit S. Darlot F. Stamegna J. C. Sanchez P. Gauthier P. Kastner A. (2008). Long-term reorganization of respiratory pathways after partial cervical spinal cord injury. *Eur. J. Neurosci.* 27 897–908. 10.1111/j.1460-9568.2008.06072.x 18279359

[B206] Vinit S. Gauthier P. Stamegna J. C. Kastner A. (2006). High cervical lateral spinal cord injury results in long-term ipsilateral hemidiaphragm paralysis. *J. Neurotrauma* 23 1137–1146. 10.1089/neu.2006.23.1137 16866626

[B207] Vinit S. Kastner A. (2009). Descending bulbospinal pathways and recovery of respiratory motor function following spinal cord injury. *Respir. Physiol. Neurobiol.* 169 115–122. 10.1016/j.resp.2009.08.004 19682608

[B208] Vinit S. Lovett-Barr M. R. Mitchell G. S. (2009). Intermittent hypoxia induces functional recovery following cervical spinal injury. *Respir. Physiol. Neurobiol.* 169 210–217. 10.1016/j.resp.2009.07.023 19651247 PMC2783733

[B209] Vinit S. Stamegna J. C. Boulenguez P. Gauthier P. Kastner A. (2007). Restorative respiratory pathways after partial cervical spinal cord injury: role of ipsilateral phrenic afferents. *Eur. J. Neurosci.* 25 3551–3560. 10.1111/j.1460-9568.2007.05619.x 17610574

[B210] Wakai J. Takamura D. Morinaga R. Nakamuta N. Yamamoto Y. (2015). Differences in respiratory changes and Fos expression in the ventrolateral medulla of rats exposed to hypoxia, hypercapnia, and hypercapnic hypoxia. *Respir. Physiol. Neurobiol.* 215 64–72. 10.1016/j.resp.2015.05.008 26001678

[B211] Ward P. J. Herrity A. N. Smith R. R. Willhite A. Harrison B. J. Petruska J. C. (2014). Novel multi-system functional gains via task specific training in spinal cord injured male rats. *J. Neurotrauma* 31 819–833. 10.1089/neu.2013.3082 24294909 PMC3996943

[B212] Warren P. M. Alilain W. J. (2014). The challenges of respiratory motor system recovery following cervical spinal cord injury. *Prog. Brain Res.* 212 173–220. 10.1016/B978-0-444-63488-7.00010-0 25194199

[B213] Warren P. M. Steiger S. C. Dick T. E. Macfarlane P. M. Alilain W. J. Silver J. (2018). Rapid and robust restoration of breathing long after spinal cord injury. *Nat. Commun.* 9:4843. 10.1038/s41467-018-06937-0 30482901 PMC6258702

[B214] Weishaupt N. Blesch A. Fouad K. (2012). BDNF: the career of a multifaceted neurotrophin in spinal cord injury. *Exp. Neurol.* 238 254–264. 10.1016/j.expneurol.2012.09.001 22982152

[B215] Weishaupt N. Li S. Di Pardo A. Sipione S. Fouad K. (2013). Synergistic effects of BDNF and rehabilitative training on recovery after cervical spinal cord injury. *Behav. Brain Res.* 239 31–42. 10.1016/j.bbr.2012.10.047 23131414

[B216] Welch J. F. (2021). Intermittent hypercapnic hypoxia: a model to study human respiratory motor plasticity?. *J. Physiol.* 599 1951–1953. 10.1113/JP281129 33480443

[B217] Welch J. F. Perim R. R. Argento P. J. Sutor T. W. Vose A. K. Nair J. (2021). Effect of acute intermittent hypoxia on cortico-diaphragmatic conduction in healthy humans. *Exp. Neurol.* 339:113651. 10.1016/j.expneurol.2021.113651 33607080 PMC8678369

[B218] Wen M. H. Lee K. Z. (2018). Diaphragm and Intercostal Muscle Activity after Mid-Cervical Spinal Cord Contusion in the Rat. *J. Neurotrauma* 35 533–547. 10.1089/neu.2017.5128 28844175

[B219] Wen M. H. Wu M. J. Vinit S. Lee K. Z. (2019). Modulation of Serotonin and Adenosine 2A Receptors on Intermittent Hypoxia-Induced Respiratory Recovery following Mid-Cervical Contusion in the Rat. *J. Neurotrauma* 36 2991–3004. 10.1089/neu.2018.6371 31099299

[B220] Wilkerson J. E. Mitchell G. S. (2009). Daily intermittent hypoxia augments spinal BDNF levels, ERK phosphorylation and respiratory long-term facilitation. *Exp. Neurol.* 217 116–123. 10.1016/j.expneurol.2009.01.017 19416672 PMC2691872

[B221] Wilkerson J. E. R. Devinney M. Mitchell G. S. (2018). Intermittent but not sustained moderate hypoxia elicits long-term facilitation of hypoglossal motor output. *Respir. Physiol. Neurobiol.* 256 15–20. 10.1016/j.resp.2017.10.005 29074449 PMC6768075

[B222] Winslow C. Rozovsky J. (2003). Effect of spinal cord injury on the respiratory system. *Am. J. Phys. Med. Rehabil.* 82 803–814. 10.1097/01.PHM.0000078184.08835.01 14508412

[B223] Wu M. J. Vinit S. Chen C. L. Lee K. Z. (2020). 5-HT7 Receptor Inhibition Transiently Improves Respiratory Function Following Daily Acute Intermittent Hypercapnic-Hypoxia in Rats With Chronic Midcervical Spinal Cord Contusion. *Neurorehabil. Neural Repair* 34 333–343. 10.1177/1545968320905806 32102596

[B224] Xie H. Leung K. L. Chen L. Chan Y. S. Ng P. C. Fok T. F. (2010). Brain-derived neurotrophic factor rescues and prevents chronic intermittent hypoxia-induced impairment of hippocampal long-term synaptic plasticity. *Neurobiol. Dis.* 40 155–162. 10.1016/j.nbd.2010.05.020 20553872

[B225] Zholudeva L. V. Abraira V. E. Satkunendrarajah K. Mcdevitt T. C. Goulding M. D. Magnuson D. S. K. (2021). Spinal Interneurons as Gatekeepers to Neuroplasticity after Injury or Disease. *J. Neurosci.* 41 845–854. 10.1523/JNEUROSCI.1654-20.2020 33472820 PMC7880285

[B226] Zholudeva L. V. Lane M. A. (2018). Choosing the right cell for spinal cord repair. *J. Neurosci. Res*. 97 109–111. 10.1002/jnr.24351 30383302 PMC6430146

[B227] Zholudeva L. V. Lane M. A. (2019). Transplanting Cells for Spinal Cord Repair: who, What, When, Where and Why?. *Cell Transplant.* 28 388–399. 10.1177/0963689718824097 30654638 PMC6628562

[B228] Zholudeva L. V. Qiang L. Marchenko V. Dougherty K. J. Sakiyama-Elbert S. E. Lane M. A. (2018). The Neuroplastic and Therapeutic Potential of Spinal Interneurons in the Injured Spinal Cord. *Trends Neurosci.* 41 625–639. 10.1016/j.tins.2018.06.004 30017476 PMC6109421

[B229] Zhou S. Y. Basura G. J. Goshgarian H. G. (2001a). Serotonin(2) receptors mediate respiratory recovery after cervical spinal cord hemisection in adult rats. *J. Appl. Physiol.* 91 2665–2673. 10.1152/jappl.2001.91.6.2665 11717232

[B230] Zhou S. Y. Castro-Moure F. Goshgarian H. G. (2001b). Activation of a latent respiratory motor pathway by stimulation of neurons in the medullary chemoreceptor area of the rat. *Exp. Neurol.* 171 176–184. 10.1006/exnr.2001.7740 11520132

[B231] Zimmer M. B. Nantwi K. Goshgarian H. G. (2007). Effect of spinal cord injury on the respiratory system: basic research and current clinical treatment options. *J. Spinal Cord Med.* 30 319–330. 10.1080/10790268.2007.11753947 17853653 PMC2031930

