# Supplementary material for: Respiratory Training and Plasticity After Cervical Spinal Cord Injury
Source: Front Cell Neurosci. 2021 Sep 21;15:700821. doi: 10.3389/fncel.2021.700821 (PMC8490715; doi:10.3389/fncel.2021.700821)
Supplement: Supplementary file 1 [file Table_1.docx]

**Table 1. Intermittent Hypoxia: Experimental Outcomes.**The following table provides a detailed summary of prior research studies exploring the use of hypoxia in pre-clinical models. 5HT: 5-hydroxytryptophan, a.k.a. serotonin; AIH: acute intermittent hypoxia; BDNF: brain derived neurotrophic factor; ChABC: chondroitinase ABC; CIH: chronic intermittent hypoxia; CPP: crossed phrenic phenomenon; dAIH: daily AIH; HIF1: hypoxia inducible factor-1; IH: intermittent hypoxia; mTOR: mammalian target of rapamycin; pLTF: phrenic long term facilitation; PTEN: phosphatase and tensin homolog; SCI: spinal cord injury; TrkB: tyrosine receptor kinase B; pTrkB: phosphorylated TrkB; VEGF: vascular endothelial growth factor.

| **Publication** | **Type of injury** | **Level** | **Model** | **Sex** | **IH protocol** | **IH applied (post-injury)** | **Duration** | **Therapeutic combination** | **Outcome** |
| --- | --- | --- | --- | --- | --- | --- | --- | --- | --- |
| (Hayashi et al., 1993) | Intact | N/A | Rat (Sprague-Dawley) | 40 Male | 3 X 5-min isocapnic hypoxia (5% O_2_) | N/A | 1 Treatment Day | N/A | Activation of chemoreceptor afferents (carotid sinus stimulation (20Hz)) and exposure to hypoxia elicits a lasting increase in phrenic nerve activity coined long-term facilitation (LTF). |
| (Bach and Mitchell, 1996) | Intact | N/A | Rat | 7 Male | 3x 5-min IH (10% O_2_) alternating with 5-min of hyperoxia (50% O_2_) | N/A | 1 Treatment day | Pretreatment with broad 5-HT antagonist Methysergide (4mg/kg) | Hypoxia-indued LTF is serotonin dependent |
| Baker 2000 | Intact | N/A | Rat (Sprague-Dawley) | 19 Male | 1) 3x3min IH 11%O_2_) with alternating hyperoxia (50% O_2_)  2) 9-Min  Or  3) 20-min of continuous hypoxia (11%O_2_) | N/A | 1 Treatment day | N/A | Hypoxia-Induced LTF is pattern sensitive. Intermittent but not sustained hypoxia was able to elicit LTF. |
| (Baker-Herman and Mitchell, 2002) | Intact | N/A | Rat (Sprague Dawley) | Male | AIH  (3 x 5-min episodes of 11% O_2_) | N/A | 1 Treatment Day | Intrathecal injection methysergide or protein synthesis inhibitors (emetine or cycloheximide) in the cervical spinal cord of anesthetized rats. | AIH-induced phrenic LTF requires serotonin protein synthesis and receptor activation. |
| (Fuller et al., 2003) | Hemisection | C2 | Rat (Sprague Dawley) | 58 Male | CIH (5 min 11%O_2_, 12 h/night) | 7 days | 7 nights | N/A | Increased CPP in normoxia, hypoxia and hypercapnia, increase evoked potentials |
| (Baker-Herman et al., 2004) | Intact | N/A | Rat (Sprague Dawley) | Male | AIH  (3 x 5-min episodes of 11% O_2_)  Or  Intermittent hypercapnia (3x 5-min episodes arterial *P*CO_2_ ∼85 mmHg) | N/A | 1 Treatment Day | Intrathecal emetine (protein synthesis inhibitor) or methysergide maleate (serotonin receptor antagonist)intrathecal BDNF injections tyrosine kinase inhibitor K252a  Small interfering RNAs (siRNA) directed against BDNF mRNA or a scrambled sequence | AIH-induced synthesis of brain-derived neurotrophic factor (BDNF) is necessary and sufficient to evoke pLTF.  There was no increase in BDNF pLTF with hypercapnia exposure. |
| (Golder and Mitchell, 2005) | Hemisection | C2 | Rat | 47 Male (Sprague Dawley)  41 Male (Lewis) | AIH (3x5 min 10-12% O2) | 7 days | Acute | A2A antagonists | Increase CPP at 8 weeks post-injury, not 2 weeks. A2A antagonist prevents this recovery |
| (Doperalski and Fuller, 2006) | Hemisection | C2 | Rat (Sprague Dawley) | 19 Male | AIH (5x3 min 13-14% O2) | 7 days | Acute |  | Increase pLTF on ipsilateral not contralateral at 4- and 8-weeks post-injury |
| (Wilkerson and Mitchell, 2009) | Intact | N/A | Rat (Brown Norway) | 47 Male | dAIH (10x 5-min of 11% O_2_) | N/A | 7 Days | N/A | Daily AIH Elicits phrenic and hypoglossal LTF respiratory metaplasticity. AIH enhances BDNF expression in Sham but not dAIH pre-treated animals and increased ERK1 and ERK2 phosphorylation. |
| (Lovett-Barr et al., 2012) | Hemisection | C2 | Rat | Male  (Lewis and Sprague Dawley) | dAIH (10x5 min 10.5%O2) | 7 days | 7 days | N/A | Restore ventilation, increase ipsilateral phrenic nerve activity, restore locomotor activity (ladder walking), increase BDNF, TrkB and pTrkB in motoneurons |
| (Gutierrez et al., 2013) | Hemisection | C2 | Rat (Sprague Dawley) | Female | dAIH (6x5 min 11%O2) | 7 days | 5 days | N/A | Increase mTOR expression in phrenic motoneurons and decrease PTEN expression in phrenic motoneurons, increase c-Fos and S6 |
| (Navarrete-Opazo et al., 2014) | Hemisection | C2 | Rat (Sprague Dawley) | 28 Male | AIH (10x 5-min 10.5% O_2_, 5-min intervals of normoxia) | 1 Week (acute) or 8 weeks (chronic) | Only 1 treatment on the designated recording days. | Systemic adenosine 2aA (A2A) receptor inhibition with intraperitoneal (IP) KW6002 | AIH evokes diaphragm and second external intercostal LTF after chronic, but not acute cervical SCI. A2A receptor antagonist enhances diaphragm LTF in normal (intact) but not chronic (C2 hemisection) animals and no LTF within the external intercostal muscles. |
| (Prosser-Loose et al., 2015) | Hemisection | C2 | Rat  (Lewis) | Male | dAIH (10x5 min 11%O2) | 4 weeks | 7 days | Ladder, treadmill, reach-to-grasp training | Delayed IH treatment does not elicit more recovery. However, combination IH with locomotor training further improve recovery and demonstrate the effect of IH for task-specific training only on ladder performance |
| (Navarrete-Opazo et al., 2015) | Hemisection | C2 | Rat (Sprague Dawley) | Male | dAIH (10x5 min 10.5%O2) | 7 days | 7 days | Repetition of IH (reminders) for 4 weeks with/without  A2A receptor antagonist (KW6002) | Increase tidal volume, contralateral diaphragm and T2 intercostal activities, dependent of A2A receptors for diaphragm, not intercostals, no effects of IH in grooming behavior |
| (Turner et al., 2016) | Intact | N/A | Mouse (129SVE) | 48 Male | IH (3 × 1 min, 15% O_2_) | N/A | 1 Treatment Day | intraperitoneal injection of ampakine CX717 (15 mg/kg) 10 min before IH | Mice given the ampakine injection pretreatment before IH had enhanced LTF (when initial baseline amplitude was low). Therefore ampakine pretreatment can facilitate IH-induced respiratory plasticity (LTF). |
| (Komnenov et al., 2016) | Hemisection | C2 | Mouse  (C57BL/6-129Sv; (32 Tph2^+/+^ and 31 Tph2^−/−^)) | Male | dAIH (12x4 min 10% O2) | 4 days | 10 days | Depletion of serotonin (Tph2-/-) | Recovery of diaphragm activity following IH not dependent on 5HT, Tidal volume, partial recovery of limb movement (but not IH of 5HT dependent, that is spontaneous recovery) |
| (Satriotomo et al., 2016) | Intact | N/A | Rat (Sprague Dawley) | 20 Male | wAIH (10x5 min 10.5%O2) + 3 times a week | N/A | 3x Week for 10 weeks | N/A | Repetitive acute intermittent hypoxia (rAIH) enhances BDNF and its high-affinity receptor TrkB and vascular endothelial growth factor A (VEGA) and hypoxia-inducible factor-1α (HIF-1α) around non-respiratory alpha motor neurons within the spinal cervical (C7) and lumbar (L3) regions and in the upper motoneurons of the primary motor cortex. |
| (Navarrete-Opazo et al., 2017) | Hemisection | C2 | Rat (Sprague Dawley) | Male | dAIH (10x5 min 10.5%O2) + 3 times a week | 8 weeks | 7 days + 8 weeks | Repetition of 3 times a week for 8 weeks and A2A antagonists | Increase tidal volume, enhanced by A2A blockade, increase bilateral diaphragm activity but not T2 intercostal activity, IH effects last 4 weeks post-treatment |
| (Lee et al., 2017) | Contusion | C3-C4 | Rat (Sprague-Dawley) | 57 Male | AIH (10x5 min 10%O2, 4% CO2) | 4 weeks | Acute | N/A | An increase in tidal volume and frequency in sham and contused animals induce long-term facilitation in contused animals at 15- and 30-min post-IH treatment |
| (Wilkerson et al., 2018) | Intact | N/A | Rat (Sprague-Dawley) | 37 Male | AIH (3 x 5 min of 11% O_2_) or ASH (a single 25 min episode of 11% O_2_) | N/A | 1 Treatment day | N/A | Increased hypoglossal motor output (LTF) after intermittent (AIH), but not sustained (ASH) hypoxia but no changes in frequency LTF with either type of hypoxia exposure.  Hypoglossal LTF, like phrenic, is pattern sensitive. |
| (Dougherty et al., 2018) | Hemisection | C2 | Rat  (Sprague-Dawley and Lewis) | Male | dAIH (10x5 min 10.5%O2) | 1 week and 7 weeks | 7 days | Methysergide | dAIH restores ventilation at 2 weeks post-injury in a 5HT independent mechanism and restore breathing at 8 weeks post-injury in a serotonin dependent mechanism |
| (Hassan et al., 2018) | Hemisection | C2 | Rat  (Lewis) | 24 Male | dAIH (10x5 min 11%O2) | 4 weeks | 1 day or 7 days | Ladder training | dAIH and ladder training increase HIF1, VEGF, BDNF, TrkB and pTrkB in the cervical and lumbar part of the spinal cord |
| (Warren et al., 2018) | Hemisection | C2 | Rat  (Sprague-Dawley) | 157 Female | dAIH (10x5 min 11%O2) | 3 months to 1.5 year | 5 days | ChABC injection 1 week before dAIH | ChABC + dAIH treatment restored diaphragm function and strengthened the ventilatory response with 5HT excessive sprouting |
| (Wen et al., 2019) | Contusion | C3-C4 | Rat  (Sprague-Dawley) | 69 Male | AIH (10x5 min 10%O2, 4% CO2) | 3 days, 2 weeks, 8 weeks | Acute | Methysergide, A2A antagonist before AIH | An increase of minute ventilation is attenuated by Methysergide but enhanced with an A2A antagonist before AIH application |
| (Wu et al., 2020) | Contusion | C3-C4 | Rat  (Sprague-Dawley) | 38 Male | AIH (10x5 min 10%O2, 4% CO2) | 8 weeks | 5 days | 5HT7 antagonist | 5HT-7 antagonist improves Vt in AIH treated animals after mid-cervical contusion. |
| (Arnold et al., 2021) | Hemisection | C3 | Rat  (Sprague-Dawley) | Male | daily AIH (10, 5 min episodes of 11% inspired O_2_ (1 week)  Then reduced to 4 treatments per week for 11 weeks. | 8 weeks | 7 days (first week), then 4 days per week for 11 weeks | Pellet reaching and horizontal ladder training | Prolonged AIH combined with task-specific reaching training significantly improved forelimb reach-to-grasp function in rats with a chronic cervical hemisection, but not off-target motor tasks (ladder walking or adhesive removal). This effect demonstrates the synergistic effect of AIH with task-specific training. |
| (Ciesla et al., 2021) | Hemisection | C2 | Rat  (Sprague-Dawley) | 57  Male | 1)Daily acute (dAIH28: 10x 5 min 10.5% O_2_  2)Mild chronic IH (IH28-5/5: 5 min 10.5% O_2_ episodes; 5 min intervals; 8 h/day  3)moderate chronic IH (IH28-2/2: 2 min 10.5% O_2_ episodes; 2 min intervals; 8 h/day) | 8 weeks | 12 weeks | N/A | IH does not change the extent of serotonergic reinnervation after a C2 hemisection injury. Although there was an increase in serotonergic structure size and the total area around phrenic motoneurons in uninjured animals, but not injured. |

Arnold, B.M., Toosi, B.M., Caine, S., Mitchell, G.S., and Muir, G.D. (2021). Prolonged acute intermittent hypoxia improves forelimb reach-to-grasp function in a rat model of chronic cervical spinal cord injury. *Exp Neurol* 340**,** 113672.

Bach, K.B., and Mitchell, G.S. (1996). Hypoxia-induced long-term facilitation of respiratory activity is serotonin dependent. *Respir Physiol* 104**,** 251-260.

Baker-Herman, T.L., Fuller, D.D., Bavis, R.W., Zabka, A.G., Golder, F.J., Doperalski, N.J., Johnson, R.A., Watters, J.J., and Mitchell, G.S. (2004). BDNF is necessary and sufficient for spinal respiratory plasticity following intermittent hypoxia. *Nat Neurosci* 7**,** 48-55.

Baker-Herman, T.L., and Mitchell, G.S. (2002). Phrenic long-term facilitation requires spinal serotonin receptor activation and protein synthesis. *J Neurosci* 22**,** 6239-6246.

Ciesla, M.C., Seven, Y.B., Allen, L.L., Smith, K.N., Asa, Z.A., Simon, A.K., Holland, A.E., Santiago, J.V., Stefan, K., Ross, A., Gonzalez-Rothi, E.J., and Mitchell, G.S. (2021). Serotonergic innervation of respiratory motor nuclei after cervical spinal injury: Impact of intermittent hypoxia. *Exp Neurol* 338**,** 113609.

Doperalski, N.J., and Fuller, D.D. (2006). Long-term facilitation of ipsilateral but not contralateral phrenic output after cervical spinal cord hemisection. *Exp Neurol* 200**,** 74-81.

Dougherty, B.J., Terada, J., Springborn, S.R., Vinit, S., Macfarlane, P.M., and Mitchell, G.S. (2018). Daily acute intermittent hypoxia improves breathing function with acute and chronic spinal injury via distinct mechanisms. *Respir Physiol Neurobiol* 256**,** 50-57.

Fuller, D.D., Johnson, S.M., Olson, E.B., Jr., and Mitchell, G.S. (2003). Synaptic pathways to phrenic motoneurons are enhanced by chronic intermittent hypoxia after cervical spinal cord injury. *J Neurosci* 23**,** 2993-3000.

Golder, F.J., and Mitchell, G.S. (2005). Spinal synaptic enhancement with acute intermittent hypoxia improves respiratory function after chronic cervical spinal cord injury. *J Neurosci* 25**,** 2925-2932.

Gutierrez, D.V., Clark, M., Nwanna, O., and Alilain, W.J. (2013). Intermittent hypoxia training after C2 hemisection modifies the expression of PTEN and mTOR. *Exp Neurol* 248**,** 45-52.

Hassan, A., Arnold, B.M., Caine, S., Toosi, B.M., Verge, V.M.K., and Muir, G.D. (2018). Acute intermittent hypoxia and rehabilitative training following cervical spinal injury alters neuronal hypoxia- and plasticity-associated protein expression. *PLoS One* 13**,** e0197486.

Hayashi, F., Coles, S.K., Bach, K.B., Mitchell, G.S., and Mccrimmon, D.R. (1993). Time-dependent phrenic nerve responses to carotid afferent activation: intact vs. decerebellate rats. *Am J Physiol* 265**,** R811-819.

Komnenov, D., Solarewicz, J.Z., Afzal, F., Nantwi, K.D., Kuhn, D.M., and Mateika, J.H. (2016). Intermittent hypoxia promotes recovery of respiratory motor function in spinal cord-injured mice depleted of serotonin in the central nervous system. *J Appl Physiol (1985)* 121**,** 545-557.

Lee, K.Z., Chiang, S.C., and Li, Y.J. (2017). Mild Acute Intermittent Hypoxia Improves Respiratory Function in Unanesthetized Rats With Midcervical Contusion. *Neurorehabil Neural Repair* 31**,** 364-375.

Lovett-Barr, M.R., Satriotomo, I., Muir, G.D., Wilkerson, J.E., Hoffman, M.S., Vinit, S., and Mitchell, G.S. (2012). Repetitive intermittent hypoxia induces respiratory and somatic motor recovery after chronic cervical spinal injury. *J Neurosci* 32**,** 3591-3600.

Navarrete-Opazo, A., Dougherty, B.J., and Mitchell, G.S. (2017). Enhanced recovery of breathing capacity from combined adenosine 2A receptor inhibition and daily acute intermittent hypoxia after chronic cervical spinal injury. *Exp Neurol* 287**,** 93-101.

Navarrete-Opazo, A., Vinit, S., Dougherty, B.J., and Mitchell, G.S. (2015). Daily acute intermittent hypoxia elicits functional recovery of diaphragm and inspiratory intercostal muscle activity after acute cervical spinal injury. *Exp Neurol* 266**,** 1-10.

Navarrete-Opazo, A.A., Vinit, S., and Mitchell, G.S. (2014). Adenosine 2A receptor inhibition enhances intermittent hypoxia-induced diaphragm but not intercostal long-term facilitation. *J Neurotrauma* 31**,** 1975-1984.

Prosser-Loose, E.J., Hassan, A., Mitchell, G.S., and Muir, G.D. (2015). Delayed Intervention with Intermittent Hypoxia and Task Training Improves Forelimb Function in a Rat Model of Cervical Spinal Injury. *J Neurotrauma* 32**,** 1403-1412.

Satriotomo, I., Nichols, N.L., Dale, E.A., Emery, A.T., Dahlberg, J.M., and Mitchell, G.S. (2016). Repetitive acute intermittent hypoxia increases growth/neurotrophic factor expression in non-respiratory motor neurons. *Neuroscience* 322**,** 479-488.

Turner, S.M., Elmallah, M.K., Hoyt, A.K., Greer, J.J., and Fuller, D.D. (2016). Ampakine CX717 potentiates intermittent hypoxia-induced hypoglossal long-term facilitation. *J Neurophysiol* 116**,** 1232-1238.

Warren, P.M., Steiger, S.C., Dick, T.E., Macfarlane, P.M., Alilain, W.J., and Silver, J. (2018). Rapid and robust restoration of breathing long after spinal cord injury. *Nat Commun* 9**,** 4843.

Wen, M.H., Wu, M.J., Vinit, S., and Lee, K.Z. (2019). Modulation of Serotonin and Adenosine 2A Receptors on Intermittent Hypoxia-Induced Respiratory Recovery following Mid-Cervical Contusion in the Rat. *J Neurotrauma* 36**,** 2991-3004.

Wilkerson, J.E., and Mitchell, G.S. (2009). Daily intermittent hypoxia augments spinal BDNF levels, ERK phosphorylation and respiratory long-term facilitation. *Exp Neurol* 217**,** 116-123.

Wilkerson, J.E.R., Devinney, M., and Mitchell, G.S. (2018). Intermittent but not sustained moderate hypoxia elicits long-term facilitation of hypoglossal motor output. *Respir Physiol Neurobiol* 256**,** 15-20.

Wu, M.J., Vinit, S., Chen, C.L., and Lee, K.Z. (2020). 5-HT7 Receptor Inhibition Transiently Improves Respiratory Function Following Daily Acute Intermittent Hypercapnic-Hypoxia in Rats With Chronic Midcervical Spinal Cord Contusion. *Neurorehabil Neural Repair***,** 1545968320905806.
