# Supplementary material for: Respiratory Training and Plasticity After Cervical Spinal Cord Injury
Source: Front Cell Neurosci. 2021 Sep 21;15:700821. doi: 10.3389/fncel.2021.700821 (PMC8490715; doi:10.3389/fncel.2021.700821)
Supplement: Supplementary file 2 [file Table_2.docx]

**Table 2. Intermittent Hypoxia: Clinical Outcomes.** The following table provides a detailed summary of prior research studies using hypoxia in clinical research studies. 6MWT: 6-Minute Walk Test; 9-HPT: Nine Hole Peg Test; 10MWT: 10-Meter Walk Test; AIH: acute intermittent hypoxia; BBT: Box and Block Test; BWSTT: Bodyweight supported treadmill training; C: cervical; dAIH: daily AIH; EMG: electromyography; FDI: first dorsal interosseous; L: lumbar; LTF: long term facilitation; MEPs: motor evoked potentials; PCMS: Paired corticospinal-motoneuronal stimulation; PNS: peripheral nerve stimulation; T: thoracic; TMS: transcranial magnetic stimulation.

| **Publications** | **Level** | **ASIA Score** | **Sex** | **Age** | **IH applied (post-injury)** | **IH protocol** | **Duration** | **Therapeutic combination** | **Outcome** |
| --- | --- | --- | --- | --- | --- | --- | --- | --- | --- |
| (Trumbower, Jayaraman et al. 2012) | C5-T7 | 5 C  8 D | 1= Female  12= Male | 46+/- 11 | 10 years (+- 7) | AIH (15x60 seconds 9%O2 or 15x90 seconds 9%O2) | 1 Day |  | AIH increase plantarflexion torque immediately and last 90 min post-treatment, correlated with EMG |
| (Tester, Fuller et al. 2014) | C3-T10 | 1 A  2 C  5 D | 4 Female  4 Male | 54.1 +/- 10.9 | 5.1 year (+- 1.7) | AIH (+2mmHg CO2 above resting values, and 8x2 min 8%O2, with a boost of 100%O2 for one breath following each hypoxic episode) | Acute -10 days, 5 days a week |  | Robust increase in minute ventilation, but no effect on ventilatory long term facilitation |
| (Hayes, Jayaraman et al. 2014) | C2-T8 | 2 C  17 D= | 3= Female  16= Male | 43 +/-4 | 9 years (+-2) | dAIH (15x90 seconds 9%O2) | 1 day and 5 days | 30 min daily walking | dAIH improved walking speed and endurance, and this effect is enhanced with a combination of daily walking training |
| (Sankari, Bascom et al. 2015) | Intact and Injured | 8 Able-bodied  8 Cervical (C4–C7)  8 Thoracic (T1–T6) | Human | 8 Female  16 Male | 15 episodes of hypoxia (1 min duration- 8% O_2_, and 40% CO_2_ to achieve oxygen saturation ≤90%) followed by room air (RA)) followed by exposure (2-3 min) to room air (RA) | Chronic  (>1 yr after injury) | 2 trails – AIH and Sham conducted on different days around the same time of day. | N/A | Individuals with cervical SCI, but not thoracic SCI, had ventilatory LTF after AIH (with sustained CO_2_) when compared with able-bodied individuals. |
| (Jaiswal, Tester et al. 2016) | C4 | D | 1 Female  (case study) | 55 | 4.7 years (chronic) (case study) | AIH (+3.8mmHg CO2 above resting values, and 8x2 min 9%O2, with a boost of 100%O2 for one breath following each hypoxic episode) | 10 days |  | Significant improvements in airflow generated in response to the applied inspiratory resistive load |
| (Navarrete-Opazo, Alcayaga et al. 2016) | C4-L4 | 13 C  22 D | 4 Female  31 Male | 41+/- 17 | Chronic > 2 years | dAIH (15x90 seconds 9%O2) for 5 days + 3 times a week | 5 days + 3 times a week | Bodyweight supported treadmill training  (BWSTT) | IH treatment plus BWSTT have a better verbal memory performance, but no difference in other cognitive test have been shown - no memory impairment |
| (Navarrete-Opazo, Alcayaga et al. 2017) | C4-L4 | 13 C  22 D | 4 Female  31 Male | 41+/- 17 | Chronic > 1 year  (4.42 +/-3.33) | dAIH (15x90 seconds 9%O2) for 5 days + 3 times a week | 5 days + 3 times a week | Bodyweight supported treadmill training (BWSTT) | dAIH plus BWSTT enhance walking speed and walking endurance, and the IH repetition maintain this effect to 2 weeks post-treatment |
| (Navarrete-Opazo, Alcayaga et al. 2017) | C4-L4 | 13 C  22 D | 4 Female  31 Male | 41+/- 17 | Chronic > 1 year  (4.3 +/-3.41) | dAIH (15x90 seconds 9%O2) for 5 days + 3 times a week | 5 days + 3 times a week | Bodyweight supported treadmill training  (BWSTT) | dAIH plus BWSTT improve dynamic but not standing balance in patients with chronic SCI |
| (Lynch, Duffell et al. 2017) | C2-T12 | 10 D | 1 Female  9 Males | 51.1+/- 13.1 years | 7.7 years (+-6.3) | AIH (15x90 seconds 9%O2) | 1 day | Ibuprofen pre-IH | Increased torque after AIH (30- and 60-min post-treatment, but ibuprofen does nothing to this increase, but EMG does not increase, whereas the correlation between torque and EMG is strong in IH treated humans |
| (Trumbower, Hayes et al. 2017) | C5 | 3 C  3 D | 6 Male | 43+/- 5 | 19 years +- 1 | dAIH (15x90 seconds 9%O2) | 5 days | 20 repetition of hand opening following dAIH | Combination improved hand dexterity, function and maximum hand opening and hand EMG activities |
| (Sandhu, Gray et al. 2019) | C1-L4 | 5 C  9 D | 1 Female  13 Male | 46 | 8.5 years +-6 | AIH (15x60 seconds 9%O2) | 1 day | Prednisolone administered 1 hour prior AIH | Combination with prednisolone increased the capacity of AIH-induced motor improvement (Max plantar flexion ankle torque, Soleus EMG increased) |
| (Sandhu, Perez et al. 2021) | C4-C7 | 7 C  7 D | 2 Females  12 Males | 46.9 ± 11.9 years | chronic injury (≥6 months) | AIH (15, 60-s alternating episodes of inspiring hypoxic air (~9% O_2_) with ambient room air (20.9% O_2_)).  Sham AIH consisted of episodes of normoxia | Acute  One or two treatment days  (Outcome measures every 30 min for up to 5 h and at 24 h post-intervention). | Grip strength and pinch strength, Nine Hole Peg Test (9-HPT), and Box and Block Test (BBT). | AIH significantly improves upper limb function (grip and pinch strength) as assessed by clinical measures (i.e., Box and Block Test).  The effect of AIH peaks around 3 h post-intervention |
| (Christiansen, Chen et al. 2021) | C1-C7 | 3 A  8 C  5 D | 12 Males  4 Females | 50.2 ± 12.8 | chronic SCI (≥1 year) (8.5 years average) | AIH  15 rounds of 1 minute exposure of (9% O_2_) and ambient air (20.9% O_2_) | 1 day | Paired corticospinal-motoneuronal stimulation (PCMS) +AIH and sham AIH (PCMS+sham-AIH).  PCMS was delivered with 180 pairs of stimuli delivered every 10 s (0.1 Hz) using transcranial magnetic stimulation (TMS) over the hand representation of the primary motor cortex and supramaximal peripheral nerve stimulation (PNS) of the ulnar nerve | Both protocols increased electromyographic activity in the first dorsal interosseous (FDI) and motor evoked potentials, but there was greater activation with the AIH protocol. Therefore, PCMS effects on spinal synapses of hand motoneurons can be potentiated by AIH. |
| (Tan, Sohn et al. 2021) | C4-T9 | Did not specify | 2= Female  9= Male | 20-68 | 1-26 years | AIH (15, 90-s episodes of 10.0% O_2_ with 60s intervals at 20.9% O_2_) or SHAM (15, 90s episodes at 20.9% O_2_ with 60s intervals at 20.9% O_2_) | Acute – Daily (5 consecutive days) | 30-min of overground walking practice  10-Meter Walk Test (10MWT) and 6-Minute Walk Test (6MWT) | Participants improved overground walking performance (speed and endurance) after daily AIH + WALK, but not SHAM+WALK or interlimb coordination |
| (Welch, Perim et al. 2021) | Intact | N/A | 4 Female  7 Males | 29 +/- 6 | N/A | AIH (15 x one-minute episodes of 9% O_2_ with 1-minute room air) | N/A | Transcranial and cervical magnetic stimulation. | AIH does not enhance diaphragm motor evoked potentials (MEPs) or produced diaphragm LTF in healthy humans. |
| (Sutor, Cavka et al. 2021) | C4-T12 | 6 A  7 B  3 C  1D | 4 Females  13 Males | 34.1 +/- 14.5 years | 5.25 +/- 8.3 years | AIH (15 x 1-min episodes 10.3% O_2_); sham: 21% O_2_) | Two Sessions: AIH and Sham at least 7 days apart | N/A | A single session of AIH increased the maximal inspiratory pressure generation, but no other respiratory outcome measures. |

Christiansen, L., B. Chen, Y. Lei, M. A. Urbin, M. S. A. Richardson, M. Oudega, M. Sandhu, W. Z. Rymer, R. D. Trumbower, G. S. Mitchell and M. A. Perez (2021). "Acute intermittent hypoxia boosts spinal plasticity in humans with tetraplegia." Exp Neurol **335**: 113483.

Hayes, H. B., A. Jayaraman, M. Herrmann, G. S. Mitchell, W. Z. Rymer and R. D. Trumbower (2014). "Daily intermittent hypoxia enhances walking after chronic spinal cord injury: a randomized trial." Neurology **82**(2): 104-113.

Jaiswal, P. B., N. J. Tester and P. W. Davenport (2016). "Effect of acute intermittent hypoxia treatment on ventilatory load compensation and magnitude estimation of inspiratory resistive loads in an individual with chronic incomplete cervical spinal cord injury." J Spinal Cord Med **39**(1): 103-110.

Lynch, M., L. Duffell, M. Sandhu, S. Srivatsan, K. Deatsch, A. Kessler, G. S. Mitchell, A. Jayaraman and W. Z. Rymer (2017). "Effect of acute intermittent hypoxia on motor function in individuals with chronic spinal cord injury following ibuprofen pretreatment: A pilot study." J Spinal Cord Med **40**(3): 295-303.

Navarrete-Opazo, A., J. Alcayaga, O. Sepulveda, E. Rojas and C. Astudillo (2017). "Repetitive Intermittent Hypoxia and Locomotor Training Enhances Walking Function in Incomplete Spinal Cord Injury Subjects: A Randomized, Triple-Blind, Placebo-Controlled Clinical Trial." J Neurotrauma **34**(9): 1803-1812.

Navarrete-Opazo, A., J. Alcayaga, D. Testa and A. L. Quinteros (2016). "Intermittent Hypoxia Does not Elicit Memory Impairment in Spinal Cord Injury Patients." Arch Clin Neuropsychol **31**(4): 332-342.

Navarrete-Opazo, A., J. J. Alcayaga, O. Sepulveda and G. Varas (2017). "Intermittent Hypoxia and Locomotor Training Enhances Dynamic but Not Standing Balance in Patients With Incomplete Spinal Cord Injury." Arch Phys Med Rehabil **98**(3): 415-424.

Sandhu, M. S., E. Gray, M. Kocherginsky, A. Jayaraman, G. S. Mitchell and W. Z. Rymer (2019). "Prednisolone Pretreatment Enhances Intermittent Hypoxia-Induced Plasticity in Persons With Chronic Incomplete Spinal Cord Injury." Neurorehabil Neural Repair **33**(11): 911-921.

Sandhu, M. S., M. A. Perez, M. Oudega, G. S. Mitchell and W. Z. Rymer (2021). "Efficacy and time course of acute intermittent hypoxia effects in the upper extremities of people with cervical spinal cord injury." Exp Neurol **342**: 113722.

Sutor, T., K. Cavka, A. K. Vose, J. F. Welch, P. Davenport, D. D. Fuller, G. S. Mitchell and E. J. Fox (2021). "Single-session effects of acute intermittent hypoxia on breathing function after human spinal cord injury." Exp Neurol **342**: 113735.

Tan, A. Q., W. J. Sohn, A. Naidu and R. D. Trumbower (2021). "Daily acute intermittent hypoxia combined with walking practice enhances walking performance but not intralimb motor coordination in persons with chronic incomplete spinal cord injury." Exp Neurol **340**: 113669.

Tester, N. J., D. D. Fuller, J. S. Fromm, M. R. Spiess, A. L. Behrman and J. H. Mateika (2014). "Long-term facilitation of ventilation in humans with chronic spinal cord injury." Am J Respir Crit Care Med **189**(1): 57-65.

Trumbower, R. D., H. B. Hayes, G. S. Mitchell, S. L. Wolf and V. A. Stahl (2017). "Effects of acute intermittent hypoxia on hand use after spinal cord trauma: A preliminary study." Neurology **89**(18): 1904-1907.

Trumbower, R. D., A. Jayaraman, G. S. Mitchell and W. Z. Rymer (2012). "Exposure to acute intermittent hypoxia augments somatic motor function in humans with incomplete spinal cord injury." Neurorehabil Neural Repair **26**(2): 163-172.

Welch, J. F., R. R. Perim, P. J. Argento, T. W. Sutor, A. K. Vose, J. Nair, G. S. Mitchell and E. J. Fox (2021). "Effect of acute intermittent hypoxia on cortico-diaphragmatic conduction in healthy humans." Exp Neurol **339**: 113651.
