# Supplementary material for: Respiratory Training and Plasticity After Cervical Spinal Cord Injury
Source: Front Cell Neurosci. 2021 Sep 21;15:700821. doi: 10.3389/fncel.2021.700821 (PMC8490715; doi:10.3389/fncel.2021.700821)
Supplement: Supplementary file 3 [file Table_3.docx]

**Table 3. Hypercapnia as a respiratory stimulant and potential training strategy.** The following table provides a detailed summary of prior research studies exploring the use of hypercapnia in pre-clinical and clinical models. AIH: acute intermittent hypoxia; LTD: long term depression; LTF: long term facilitation; SCI: spinal cord injury; vLTF: ventilatory long-term facilitation.

| **Publications** | **Type of Injury** | **Level** | **Model** | **Sex** | **Protocol** | **Treatment Applied Post- Injury** | **Duration** | **Therapeutic combination** | **Outcome** |
| --- | --- | --- | --- | --- | --- | --- | --- | --- | --- |
| (Engwall, Vidruk et al. 1988) | Intact | N/A | Goat | Did not specify | Prolonged exposure to normoxic- hypercapnia (30-85 Torr PaCO_2_) for up to 240 minutes. | N/A | 1 Treatment Day | N/A | There was elevated discharge frequency of the carotid chemoreceptor after prolonged hypercapnia exposure (up to 240min) in no time-dependent manner.  This is in contrast to the time-dependent increase in carotid body activity with prolonged normocapnic-hypoxia. |
| (Teppema, Berkenbosch et al. 1994) | Intact | N/A | Cats | Did not specify | 10% CO_2_ for 1 hour | N/A | 1 Treatment Day | N/A | Increased c-fos expression within the retrotrapezoid nucleus (RTN) of the animals exposed to CO_2._ This indicates that the RTN neurons are activated during hypercapnia. |
| (Morris, Arata et al. 1996) | Intact | N/A | Cats | Not mentioned | Animals were injected with 200 ul of CO_2_ Saturated Saline (0.9%) for 30 Sec into the external carotid artery. | N/A | 1 Treatment Day | N/A | LTF after injection of CO_2_ Saturated Saline and increased firing rate of raphe neurons suggesting parallel routes by which carotid chemoreceptors influence medullary raphe neurons. Supports the hypotheses that mid-line respiratory-related neuronal assemblies transform information from those receptors and regulate the gain of respiratory motor output. |
| (Teppema, Veening et al. 1997) | Intact | N/A | Rat | Did not specify | 2 hour exposure to (a) air ; (b) 8% CO_2_  in air; (c) 10% CO_2_  in air; (d) 15% CO_2_  in air; (e) 15% CO_2_ + 60%  O_2_  (f) 9% O_2_ | N/A | 1 Treatment Day |  | Hypercapnia exposure increases c-fos expression within the brainstem (including the caudal nucleus tractus solitarius (NTS), caudal ventrolateral medulla, raphe, the retrotrapezoid nucleus (RTN); locus coeruleus (LC) etc.) in a dose-dependent manner and had greater activation than hypoxia.   Interestingly there was an increase in c-fos in respiratory and non-respiratory brainstem regions. |
| (Bach and Mitchell 1998) | Intact | N/A | Rat | 27 Male | 3x5 minute episodes of 10% CO_2_  And 3x5 minute episodes of 3-5% CO_2_ | N/A | 1 day | rats pretreated with the α2-adrenergic antagonists (yohimbine HCl and imidazoline (RX-821002) HCl) | 10% Hypercapnia induced long-term depression (LTD) in phrenic and hypoglossal neurogram recordings. This effect was attenuated with α2-adrenergic antagonist delivery |
| (Baker, Fuller et al. 2001) | Intact | N/A | Rat | Did not Specify | 25 min of approximately 10% inspired CO_2_ | N/A | 1 day | N/A | Continuous, severe  hypercapnia (25 min of approximately 10% inspired CO _2_) elicits long-term depression (LTD) of phrenic motor output. However, episodic exposure of 3x5 min hypercapnic episodes does not elicit LTD. |
| (Harris, Balasubramaniam et al. 2006) | Intact | N/A | Human | 4 Females  7 Males | carbon dioxide was sustained 5 mmHg above baseline levels during 8x 4-min episodes of 8% hypoxia | N/A | 1 Trial (some subjects came back for other trials but not all) | N/A | Sustained elevated CO_2_ is essential to induce long-term ventilatory facilitation in humans with hypoxic exposure. Did not cause vLTF with hypoxia or hypercapnia alone. |
| (Diep, Khan et al. 2007) | Intact | N/A | Human | 3 Female  7 Male | Fifteen, 30-s episodes breathing 6% O _2_ and 5% CO _2_ separated by 90 s of breathing air | N/A | 1 Treatment | N/A | No LTF or persistent increase in ventilation or decrease in end-tidal CO_2._ No ventilatory LTF in humans with patterned hypercapnic-hypoxia stimuli. |
| (Griffin, Pugh et al. 2012) | Intact | N/A | Human | 12 Males (average age 23.5 +/- 0.42 years) | 8x4 minute IH  Or continuous IH for 32 minutes  Or Euoxia | N/A | 3 Trials >24 hours apart | N/A | When CO2 is maintained above normal levels in awake humans, exposure to acute intermittent hypoxia causes a sustained elevation in ventilation that persists after normoxic breathing is resumed. |
| (Valic, Pecotic et al. 2016) | Intact | N/A | Rats (Sprague Dawley) | 39 Males | 5X 3 minutes of AIHc (15% CO_2_) alternating with 3 minutes of normoxia | N/A | 1 Day, 5 exposures | 1) i.v. injection of broad 5-HT receptor antagonist methysergide 2) i.v. injection of WAY-100635 (selective 5-HT_1A_ receptor antagonist)  3) control group received i.v. injection of saline.  20 min before exposure to intermittent hypercapnia. | Animals pre-treated with a selective 5-HT 1 antagonist prevented frequency pLTD.  Long-term decrease in phrenic nerve frequency was evoked in control and methysergide-pretreated animals.  5-HT receptors modulate AIHc respiratory plasticity. |
| (Stipica, Pavlinac Dodig et al. 2016) | Intact | N/A | Rats (Sprague-Dawley) | 46 Male | 5x 3-min intervals of:  hypercapnia (15 % CO_2_ in air),  hypoxia (9 % O_2_ in N_2_)  Hypercapnia and hypoxia (15 % CO_2_ and 9 % O_2_ in N_2_)  Separated by 3 minutes of recovery.  Two continuous groups with 30-min exposures to hypercapnia (CHc; 15 % CO_2_) or hypoxia (CH; 9 % O_2_ in N_2_) | 1 Treatment Day | N/A | N/A | Exposure to acute intermittent hypercapnia (AIHc) elicited decreased phrenic nerve frequency (frequency LTD).  Exposure to AIH elicited pLTF.  Combined AIHC and IH or continuous hypoxia (CH) and continuous hypercapnia (CHc) did not induce pLTF.  This indicates that intermittent exposures are necessary to evoke distinct forms of respiratory plasticity. |
| (Bascom, Sankari et al. 2016) | Contusion and Intact | 6 Cervical 6 Thoracic 12 Able-bodied | Human | 15 Females  39 Male | A single breath of 8–10% inspired CO_2_. Trials were performed three times with 2–3 min between trials. | Chronic | 1 day |  | Individuals with SCI had a greater ventilatory response to a single breath of CO_2_ than able-bodied controls. No difference in response based on injury level indicated greater ventilatory chemoresponsiveness to CO_2_ following SCI. |
| (Lee, Chiang et al. 2017) | Contusion | C3-C4 | Rat | 57 Male | AIH (10x5 min 10%O2, 4% CO2) | Acute | 4 weeks |  | Increased tidal volume and frequency in sham and contused animals induce long-term facilitation in contused animals at 15 and 30 min post hypoxic hypercapnic treatment |
| (Stipica Safic, Pecotic et al. 2018) | Intact | N/A | Rat (Sprague-Dawley) | 35 Male | (Acute Intermittent hypercapnia) AIHc; 5 episodes of 15% CO_2_ in air, each episode lasting 3 min | N/A | 1 Treatment day  (5 exposure) | Microinjection of the selective 5-HT_1A_ receptor agonist 8-hydroxy-2-(dipropylamino) tetralin hydrobromide (8-OH-DPAT), the broad-spectrum 5-HT antagonist methysergide, or the α2-adrenergic antagonist yohimbine, whereas the control group received microinjection of 0.9% saline into the caudal raphe region. | Peak phrenic nerve activity (pPNA) and burst frequency (*f*) were analyzed during baseline (T0), during 5 hypercapnic episodes (THc1–THc5), and at 15, 30, and 60 min after the end of the last hypercapnic episode.  60 min after AIHc leads to pLTD, which was accentuated by the activation of 5-HT1A receptors. Blockade of α2-adrenergic receptors or broad-spectrum 5-HT antagonist did not evoke LTD after AIHc. |
| (Wen, Wu et al. 2019) | Contusion | C3-C4 | Rat | 69 Male | AIH (10x5 min 10%O2, 4% CO2) | 3 days, 2 weeks, 8 weeks | 5 minutes at each time point | Methysergide, A2A antagonist before AIH | Hypercapnic-hypoxia can induce recovery from acute to chronic injury stages. Increase of minute ventilation is attenuated by methysergide (5HT antagonist) but enhanced with KW6002 (A2A antagonist) before AIH application |
| (Vermeulen, Benbaruj et al. 2020) | Intact | N/A | Human | 9 Females  10 Males (Average Age 22 +/-3) | Intermittent hypercapnic-hypoxia (40 × 40 s end-tidal O_2_ = 50 mmHg with 4 mmHg end-tidal CO_2_ above the baseline) | N/A | One treatment day (subset (n=9) returned for a follow-up within 72 hours.) |  | Hypercapnic-hypoxia evoked vLTF in healthy humans;  vLTF expression is attenuated with peripheral chemoreflex inhibition by hyperoxia. This suggests a contribution from central nervous pathways in vLTF expression;  Males and females develop vLTF through different ventilatory recruitment strategies (i.e., males have larger tidal volume, females have larger breathing frequency). |
| (Wu, Vinit et al. 2020) | Contusion | C3-C4 | Rat | 38 Male | Daily acute intermittent hypercapnic-hypoxia (10 X 5 minutes (10% O_2_, 4% CO_2)_ | 8 weeks | 5 days | 5-HT7 receptor antagonist (SB269970) delivered 5 min before treatment. | 5-HT7 receptor inhibition improved daily intermittent hypercapnic-hypoxia–induced tidal volume increase in mid-cervical spinal contused animals. |
| (Lin, Vinit et al. 2021) | Contusion | C3-C4 | Rat | 55 Male | 5-minute intervals alternating with normoxia with (1) Intermittent hypercapnic-hypoxia (10 × 5 min 10%O_2_ + 4%CO_2_  (2) Intermittent hypoxia (10 × 5 min 10%O_2_  (3) Intermittent hypercapnia (10 × 5 min 21%O_2_ + 4%CO_2_  Or  (4) Intermittent hypoxia with sustained hypercapnia (10 × 5 min 10%O_2_ + 4%CO_2_ with 5 min 21%O_2_ + 4%CO_2_ interval  (5) Sustained hypercapnia (100 min, 21% O_2_ + 4% CO_2_);  (6) Sustained normoxia (100 min, 21% O_2_). | subchronic (14 ± 1 days post-injury) and chronic (56 ± 1 days post-injury) | 1 Day | N/A | Intermittent hypoxia with hypercapnia had a greater ventilatory response than sustained hypercapnia. There was a significant increase in tidal volume with intermittent hypercapnic-hypoxia, IH with sustained hypercapnia, and IH alone at the subchronic timepoint. Only hypercapnic-hypoxia and IH with sustained hypercapnia could evoke tidal volume LTF after a chronic injury. Therefore, hypercapnia was not detrimental but did not further enhance IH effectiveness. Sustained hypercapnia can blunt the ventilatory response of IH after chronic injury. |

Bach, K. B. and G. S. Mitchell (1998). "Hypercapnia-induced long-term depression of respiratory activity requires alpha2-adrenergic receptors." J Appl Physiol (1985) **84**(6): 2099-2105.

Baker, T. L., D. D. Fuller, A. G. Zabka and G. S. Mitchell (2001). "Respiratory plasticity: differential actions of continuous and episodic hypoxia and hypercapnia." Respir Physiol **129**(1-2): 25-35.

Bascom, A. T., A. Sankari and M. S. Badr (2016). "Spinal cord injury is associated with enhanced peripheral chemoreflex sensitivity." Physiol Rep **4**(17).

Diep, T. T., T. R. Khan, R. Zhang and J. Duffin (2007). "Long-term facilitation of breathing is absent after episodes of hypercapnic hypoxia in awake humans." Respir Physiol Neurobiol **156**(2): 132-136.

Engwall, M. J., E. H. Vidruk, A. M. Nielsen and G. E. Bisgard (1988). "Response of the goat carotid body to acute and prolonged hypercapnia." Respir Physiol **74**(3): 335-344.

Griffin, H. S., K. Pugh, P. Kumar and G. M. Balanos (2012). "Long-term facilitation of ventilation following acute continuous hypoxia in awake humans during sustained hypercapnia." J Physiol **590**(20): 5151-5165.

Harris, D. P., A. Balasubramaniam, M. S. Badr and J. H. Mateika (2006). "Long-term facilitation of ventilation and genioglossus muscle activity is evident in the presence of elevated levels of carbon dioxide in awake humans." Am J Physiol Regul Integr Comp Physiol **291**(4): R1111-1119.

Lee, K. Z., S. C. Chiang and Y. J. Li (2017). "Mild Acute Intermittent Hypoxia Improves Respiratory Function in Unanesthetized Rats With Midcervical Contusion." Neurorehabil Neural Repair **31**(4): 364-375.

Lin, M. T., S. Vinit and K. Z. Lee (2021). "Functional role of carbon dioxide on intermittent hypoxia induced respiratory response following mid-cervical contusion in the rat." Exp Neurol **339**: 113610.

Morris, K. F., A. Arata, R. Shannon and B. G. Lindsey (1996). "Long-term facilitation of phrenic nerve activity in cats: responses and short time scale correlations of medullary neurones." J Physiol **490 ( Pt 2)**: 463-480.

Sankari, A., A. T. Bascom, A. Riehani and M. S. Badr (2015). "Tetraplegia is associated with enhanced peripheral chemoreflex sensitivity and ventilatory long-term facilitation." J Appl Physiol (1985) **119**(10): 1183-1193.

Stipica, I., I. Pavlinac Dodig, R. Pecotic, Z. Dogas, Z. Valic and M. Valic (2016). "Periodicity during hypercapnic and hypoxic stimulus is crucial in distinct aspects of phrenic nerve plasticity." Physiol Res **65**(1): 133-143.

Stipica Safic, I., R. Pecotic, I. Pavlinac Dodig, Z. Dogas, Z. Valic and M. Valic (2018). "Phrenic long-term depression evoked by intermittent hypercapnia is modulated by serotonergic and adrenergic receptors in raphe nuclei." J Neurophysiol **120**(1): 321-329.

Teppema, L. J., A. Berkenbosch, J. G. Veening and C. N. Olievier (1994). "Hypercapnia induces c-fos expression in neurons of retrotrapezoid nucleus in cats." Brain Res **635**(1-2): 353-356.

Teppema, L. J., J. G. Veening, A. Kranenburg, A. Dahan, A. Berkenbosch and C. Olievier (1997). "Expression of c-fos in the rat brainstem after exposure to hypoxia and to normoxic and hyperoxic hypercapnia." J Comp Neurol **388**(2): 169-190.

Valic, M., R. Pecotic, I. Pavlinac Dodig, Z. Valic, I. Stipica and Z. Dogas (2016). "Intermittent hypercapnia-induced phrenic long-term depression is revealed after serotonin receptor blockade with methysergide in anaesthetized rats." Exp Physiol **101**(2): 319-331.

Vermeulen, T. D., J. Benbaruj, C. V. Brown, B. M. Shafer, J. S. Floras and G. E. Foster (2020). "Peripheral chemoreflex contribution to ventilatory long-term facilitation induced by acute intermittent hypercapnic hypoxia in males and females." J Physiol **598**(20): 4713-4730.

Wen, M. H., M. J. Wu, S. Vinit and K. Z. Lee (2019). "Modulation of Serotonin and Adenosine 2A Receptors on Intermittent Hypoxia-Induced Respiratory Recovery following Mid-Cervical Contusion in the Rat." J Neurotrauma **36**(21): 2991-3004.

Wu, M. J., S. Vinit, C. L. Chen and K. Z. Lee (2020). "5-HT7 Receptor Inhibition Transiently Improves Respiratory Function Following Daily Acute Intermittent Hypercapnic-Hypoxia in Rats With Chronic Midcervical Spinal Cord Contusion." Neurorehabil Neural Repair: 1545968320905806.
